# A Phytochemical Perspective on Plant Defense Against Nematodes

**DOI:** 10.3389/fpls.2020.602079

**Published:** 2020-11-13

**Authors:** Willem Desmedt, Sven Mangelinckx, Tina Kyndt, Bartel Vanholme

**Affiliations:** ^1^ Research Group Epigenetics and Defense, Department of Biotechnology, Ghent University, Ghent, Belgium; ^2^ Department of Plant Biotechnology and Bioinformatics, Ghent University, Ghent, Belgium; ^3^ VIB Center for Plant Systems Biology, Ghent, Belgium; ^4^ Research Group Synthesis, Bioresources and Bioorganic Chemistry (SynBioC), Department of Green Chemistry and Technology, Ghent University, Ghent, Belgium

**Keywords:** plant-parasitic nematodes, metabolomics, secondary metabolites, nematode resistance, plant immunity, phytoalexins

## Abstract

Given the large yield losses attributed to plant-parasitic nematodes and the limited availability of sustainable control strategies, new plant-parasitic nematode control strategies are urgently needed. To defend themselves against nematode attack, plants possess sophisticated multi-layered immune systems. One element of plant immunity against nematodes is the production of small molecules with anti-nematode activity, either constitutively or after nematode infection. This review provides an overview of such metabolites that have been identified to date and groups them by chemical class (e.g., terpenoids, flavonoids, glucosinolates, etc.). Furthermore, this review discusses strategies that have been used to identify such metabolites and highlights the ways in which studying anti-nematode metabolites might be of use to agriculture and crop protection. Particular attention is given to emerging, high-throughput approaches for the identification of anti-nematode metabolites, in particular the use of untargeted metabolomics techniques based on nuclear magnetic resonance (NMR) and mass spectrometry (MS).

## Introduction

Plant-parasitic nematodes (PPN) are important agricultural pests. Although nematode parasitism is rarely fatal, PPN cause substantial yield losses by diverting nutrients, disrupting water transport, increasing susceptibility to secondary infections, and by acting as vectors for viruses (Bird and Kaloshian, 2003; Nicol et al., 2011). Although quantifying their impact is difficult, estimates suggest that PPN reduce global yields by 10–25% (Nicol et al., 2011). A single nematode species, the soybean cyst nematode *Heterodera glycines*, reduces soybean yield by nearly 10% in the United States (Savary et al., 2019).

Over four thousand PPN species have been identified (Wyss, 1997; Decraemer and Hunt, 2006; Nicol et al., 2011); the majority feed on roots, but some also feed on aerial parts (Fuller et al., 2008). Despite their diversity, most economic losses are caused by a handful of sedentary PPN genera – especially the root-knot nematodes (*Meloidogyne* spp.) and the cyst nematodes (*Heterodera* spp. and *Globodera* spp.; Fuller et al., 2008; Nicol et al., 2011). Effective nematode control is exceptionally difficult and requires an integrated approach that combines chemicals, cultural practices, biocontrol and, where available, resistant varieties (Fuller et al., 2008).

To facilitate the development of novel nematode control strategies, plant nematologists have spent considerable time and effort on studying the mechanisms of plant defense against PPN. One defense mechanism is the production of metabolites with anti-nematode activity, which in this review we will call *anti-nematode phytochemicals* (ANPs). This review provides an overview of known ANPs, discusses strategies for their identification (with particular focus on metabolomics approaches), and comments on the potential of ANPs in PPN control.

## Notes on Terminology

**Secondary Metabolite**plant metabolites can be broadly divided into two groups, primary and secondary metabolites. Primary metabolites are directly involved in the formation of new cells, whereas secondary metabolites are not required for plant growth but instead contribute to processes such as resistance to pests and diseases, attraction of pollinators, and abiotic stress tolerance (Seigler, 1998; Hartmann, 2007). This distinction is not absolute: plant hormones, lignin monomers, and various other metabolites have properties of both primary and secondary metabolites (Seigler, 1998). Furthermore, the classification of metabolites may change as plant science advances: shikimic acid and squalene were long seen as secondary metabolites but are now known to be precursors involved in the biosynthesis of primary metabolites (aromatic amino acids and sterols, respectively; Seigler, 1998). Also, “secondary” metabolites should not be seen as “non-essential”: particularly under stressful conditions, impairments in secondary metabolism are often lethal (Seigler, 1998; Hartmann, 2007). Interested readers are referred to an excellent review article (Erb and Kliebenstein, 2020) for an in-depth discussion of plant secondary metabolites and their relation to primary metabolites.

**Phytoanticipins and Phytoalexins** Biocidal secondary metabolites produced by plants as protection against pests and pathogens have been divided into *phytoanticipins* and *phytoalexins*. Phytoanticipins are defined as defense compounds which are constitutively present, i.e., regardless of the presence of pests or diseases (VanEtten et al., 1994). By contrast, phytoalexins accumulate only upon perception of pests or pathogens (VanEtten et al., 1994). However, like most distinctions in plant science, the difference between phytoanticipins and phytoalexins is not absolute. Defense compounds may be constitutively present but show a further increase in abundance after pathogen attack. They may also be constitutively present in some organs but produced only upon pest or disease induction in others. In both of those cases, the same compound is both a phytoanticipin and a phytoalexin (VanEtten et al., 1994). Furthermore, phytoalexins may be present constitutively in an inactive storage form (e.g., a glycoside) from which they are released upon pest or pathogen perception (VanEtten et al., 1994).

**Nematistatic and Nematicidal Compounds**To study the anti-nematode activity of a metabolite, researchers often expose nematodes to this metabolite in an *in vitro* assay to test whether it is directly toxic to the nematode. The most common assay involves dissolving the compound(s) of interest in water at biologically relevant concentrations and then incubating nematodes in this solution for several hours or days. If most or all nematodes become rigid and immobile, the compound is said to be *nematistatic*. At this point, the nematode may be either reversibly paralyzed or dead. To distinguish between these two possibilities, the nematodes are transferred to clean water. If nematode motility recovers, the compound is only nematistatic (paralyzing). If no recovery is seen, the nematode is dead or irreversibly paralyzed and the compound is said to be *nematicidal*. For many compounds, nematicidal and nematistatic activities are part of a spectrum: low doses and/or brief exposures might be nematistatic, whereas longer exposures or higher doses are nematicidal.

**Plant Resistance**Resistance refers to a reduced ability of a pest or pathogen to grow and reproduce on a host plant. Resistance may be qualitative, in which case disease is absent (i.e., the pest or pathogen cannot reproduce), or quantitative, in which case disease severity is reduced (i.e., the pest or pathogen can reproduce, but at a substantially lower rate than is typical for that host; St.Clair, 2010).

**Pre- and Post-penetration Resistance**Plant-parasitic nematodes resistance can be classified as *pre-penetration* or *post-penetration* resistance. Pre-penetration resistance refers to a situation in which a nematode is unable to enter the host plant due to e.g., the absence of metabolites needed for host recognition, repellent host exudates or the presence of a physical barrier the nematode is unable to penetrate (Lee et al., 2017). In post-penetration resistance, the PPN enters the host but is then unable to survive or reproduce due to e.g., the presence of toxic metabolites or an inability to feed. For sedentary PPN, this resistance can be further divided into *early* and *late* resistance; early resistance occurs during migration or early feeding site formation, whereas late resistance occurs after the nematode has established a feeding site (Fuller et al., 2008).

**Nematode Life Cycles**Plant-parasitic nematodes can be classified according to their lifestyles and modes of parasitism. *Ectoparasitic* nematodes remain outside the plant and penetrate it only with their stylet, whereas *endoparasitic* nematodes enter the host. PPN may also be classified as *sedentary* or *migratory*: migratory PPN remain motile throughout their life (e.g., *Pratylenchus* spp. and *Radophulus* spp.), whereas in sedentary PPN, only second-stage juveniles (J2s) are motile. The migratory J2 finds and penetrates a host and then forms a permanent *feeding site*, in which the nematode completes the remainder of its life cycle in a sedentary form. The principal sedentary nematodes are the *cyst nematodes* (*Heterodera* spp. and *Globodera* spp.) and the *root-knot nematodes* (*Meloidogyne* spp.). Interested readers can find a brief introduction to the life cycles of the most economically significant PPN in Jones et al. (2013), and more comprehensive discussions of the evolution, diversity, and infection mechanisms of PPN in Perry and Moens (2011) and Smant et al. (2018).

## ANPs: a Brick in the Wall of Plant Immunity

Plants possess a sophisticated system of defenses against pests and pathogens that consists of both constitutive, pre-formed mechanisms and inducible immune responses which occur upon perception of intruders.

A detailed overview of plant inducible immune responses falls outside the scope of this review (interested readers are referred to e.g., Jones and Dangl, 2006; Andolfo and Ercolano, 2015; Wang et al., 2019), but it is relevant to note that plants are capable of perceiving molecular patterns characteristic for PPN infection and display an induced immune response upon their perception. Such patterns include the pheromone ascaroside (Manosalva et al., 2015; Manohar et al., 2020) and oligogalacturonides released by the intracellular migration of certain PPN species (Sato et al., 2019). Some plants also possess dedicated resistance genes (R-genes), which encode receptors able to recognize specific PPN effectors; some of these R-genes induce a rapid, intense immune response characterized by a hypersensitive response upon PPN perception. Examples of well-characterized anti-nematode R-genes include the *Mi* genes in tomato (against several root-knot nematode species) and *Hero*, *Gpa*, and *Gro* in potato (against potato cyst nematodes; Sato et al., 2019).

In addition to induced immune responses, plants possess constitutive forms of resistance that do not necessitate pest or pathogen perception. Two examples of such resistance are *pre-penetration resistance* and *metabolic resistance*. The former refers to a situation in which a pest or pathogen cannot find or penetrate a suitable host because a molecular pattern required for host recognition is absent, or because the plant possesses an impenetrable barrier (Lee et al., 2017). Metabolic resistance occurs when a pest or pathogen attempts to penetrate a host but encounters a constitutively present metabolite that is sufficiently toxic to prevent colonization (Lee et al., 2017).

**Figure 1:**
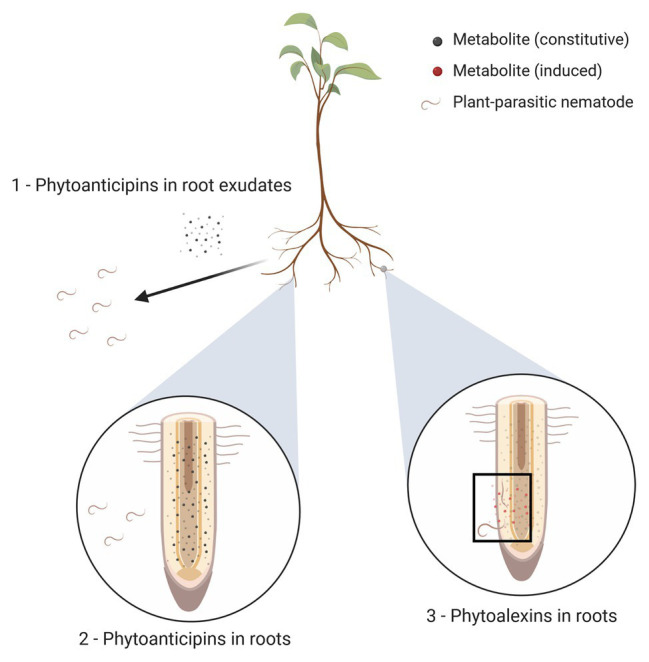
Three types of plant *anti-nematode phytochemicals* (ANPs). (1) Plant roots exude metabolites into their environments (represented here as black dots); these exudates may function as phytoanticipins by exerting repellent or nematicidal activities. (2) Plant tissues (depicted here as roots, but other plant tissues may equally be affected by nematode parasitism) may contain constitutively formed metabolites with anti-nematode activity, irrespective of actual nematode presence; these metabolites are called phytoanticipins and are represented by small black dots. (3) Upon nematode penetration, the plant may respond by locally producing additional anti-nematode compounds; these induced metabolites are called phytoalexins and are shown here as red dots present near the site of nematode penetration and migration (highlighted with black rectangle).

Both inducible and constitutive defenses against nematodes rely at least in part on the presence of secondary plant metabolites with anti-nematode activity (as shown in Figure 1), which we call ANPs in this review. The remainder of this review will focus entirely on these ANPs; for a broader overview of the various defense mechanisms plants employ against nematodes, readers are referred to other reviews (e.g., Holbein et al., 2016; Sato et al., 2019).

## An Overview of Known ANPs

Plants possess an extensive secondary metabolism capable of producing a vast diversity of metabolites; approximately 200,000 plant secondary metabolites are believed to exist (Viant et al., 2017). In this review, we have chosen to group ANPs in several classes of secondary metabolites: phenolic compounds, terpenoids, saponins, benzoxazinoids, organosulfur compounds, alkaloids, and glucosinolates. This classification is of course not the only possible one, and some compounds could be placed in more than one category.

Grouping ANP studies by chemical class also leads, to some extent, to grouping by plant family. Although all plant species produce multiple classes of secondary metabolites, each has evolved a bias toward specific classes of defensive metabolites. For example, Fabaceae defense compounds are often (iso)flavonoids, Malvaceae and Solanaceae phytoalexins are often terpenoids, and glucosinolates are unique to the order Brassicales (Veech, 1982).

This review limits itself to secondary metabolites for which there is at least tentative evidence that they are involved in plant defense against nematodes, i.e., compounds which are present in tissues affected by nematode parasitism (either constitutively or induced upon nematode infection) and whose presence could be correlated to nematode resistance. Many other nematicidal compounds have been identified in plant extracts, but without an apparent role in plant-nematode interactions; an overview of several such compounds can be found in e.g., Chitwood (2002).

### Phenolic Compounds

A major class of ANPs are those derived from the *phenylpropanoid pathway* (PPP), which are frequently lumped together under the umbrella term *phenolic compounds*. Phenolic compounds play a major role in resistance to various plant pests and diseases (Nicholson and Hammerschmidt, 1992; Lattanzio et al., 2006), and a role for phenolic compounds in nematode resistance has been suggested since at least the early 1960s. In general, higher basal and/or induced amounts of phenolic compounds have been found to correlate with nematode resistance in a wide variety of plant-nematode combinations (Wallace, 1961; Giebel, 1970, 1982; Hung and Rohde, 1973; Bajaj and Mahajan, 1977; Pegard et al., 2005; Dhakshinamoorthy et al., 2014; Hölscher et al., 2014).

Plant phenolic compounds are generally derived from the aromatic amino acids L-phenylalanine and (less commonly) L-tyrosine. L-phenylalanine is deaminated by PHENYLALANINE AMMONIA LYASE to form (*E*)-cinnamic acid, which can be *para*-hydroxylated by CINNAMIC ACID-4-HYDROXYLASE to form *para*-coumaric acid. Alternatively, *para*-coumaric acid can be formed directly through deamination of L-tyrosine by TYROSINE AMMONIA LYASE. *para*-Coumaric acid is then activated through the thioester coupling of acetyl-coenzyme A by 4-COUMARATE-CoA LIGASE to form the reactive metabolic intermediate *para*-coumaroyl-CoA. From this point onward, the PPP branches in various directions to form a dazzling array of secondary metabolites that includes hydroxycinnamic acids, flavonoids, tannins, diarylheptanoids, stilbenoids, and many others (Vogt, 2010).

Although most phenolic compounds are produced *via* the PPP, some less common phenolic metabolites, such as alkylresorcinols (Baerson et al., 2010), are produced through polyketide metabolism. These phenolic metabolites are not discussed in this review, as to the best of our knowledge they have not been studied in plant-nematode interactions.

#### Hydroxycinnamic Acids

Hydroxycinnamic acids (HAs) are hydroxy derivatives of (*E*)-cinnamic acid. The HA *para*-coumaric acid is a core intermediate in the PPP and other HAs (e.g., ferulic acid, caffeic acid, or sinapic acid) are abundant in many plants either as pure compounds or as conjugate forms (Vogt, 2010).

**Figure 2:**
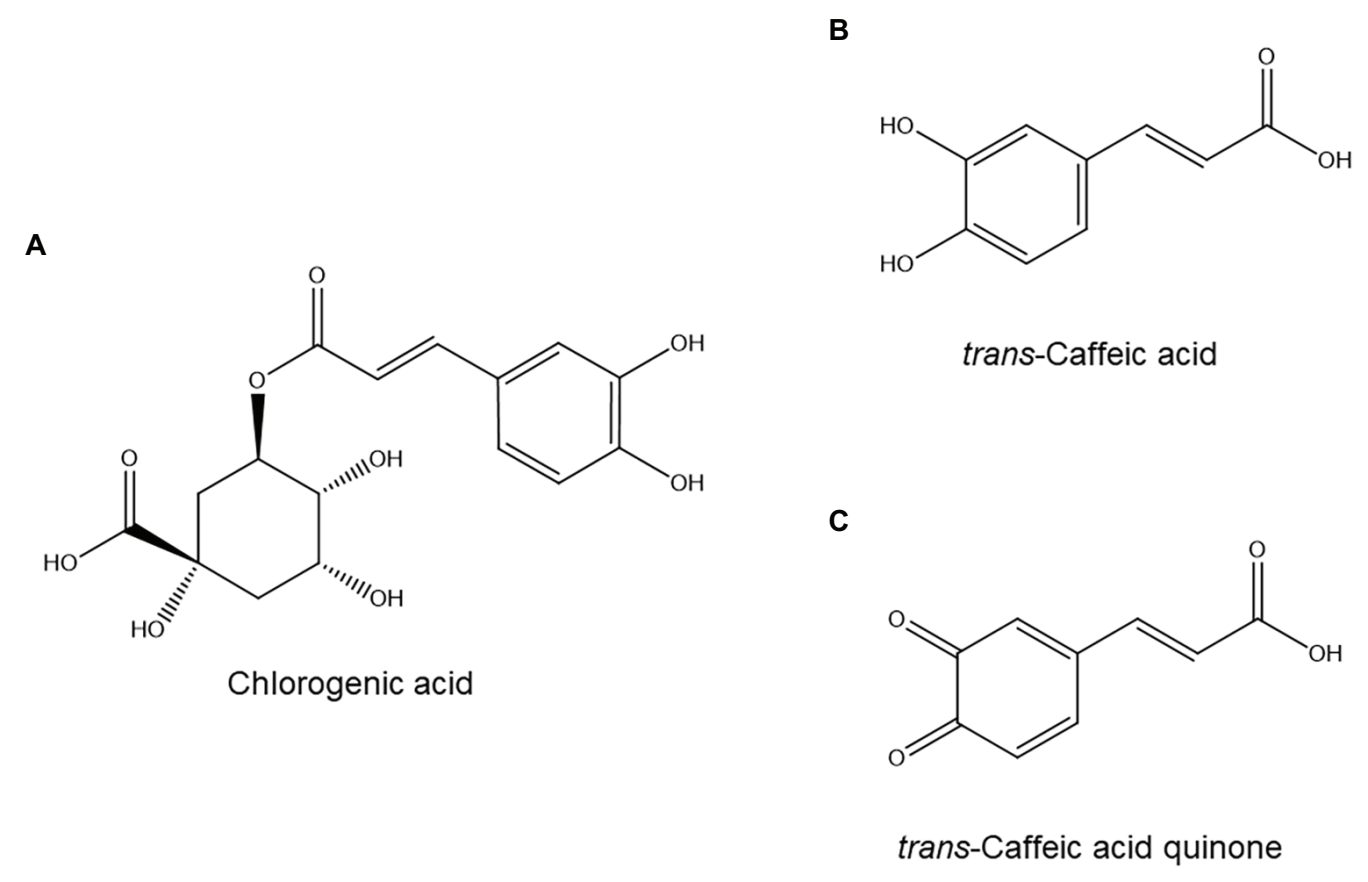
Hydroxycinnamic acids with possible roles in plant resistance to nematodes: chlorogenic acid **(A)**, caffeic acid **(B)**, and caffeic acid quinone **(C)**.

One of the first individual phenolic compounds to be implicated in defense against nematodes was chlorogenic acid (Figure 2A). This ester of caffeic acid (Figure 2B) and (-)-quinic acid accumulates in several dicot (Wallace, 1961; Hung and Rohde, 1973; Pegard et al., 2005) and monocot (Gill et al., 1996) plants in sites of PPN infection, and induced chlorogenic acid levels appear correlated to nematode resistance in various plant species (Hung and Rohde, 1973; Gill et al., 1996; Pegard et al., 2005; Meher et al., 2015). Furthermore, it was recently shown that chlorogenic acid accumulation was strongly repressed in *Meloidogyne incognita* galls in a susceptible poplar clone (*Populus tremula × Populus alba*), which suggests that suppression of chlorogenic acid biosynthesis might be a pathogenesis strategy employed by *M. incognita* (Baldacci-Cresp et al., 2020).

However, chlorogenic acid is only weakly nematicidal (Mnviajan et al., 1992; D’Addabbo et al., 2013). A plausible, but unproven, explanation for this discrepancy is that chlorogenic acid could be a precursor to an unstable, elusive nematicidal compound. One candidate is caffeic acid quinone (Figure 2C), a compound that is toxic to nematodes (Mnviajan et al., 1992) and which can be formed from chlorogenic acid: hydrolysis of chlorogenic acid affords quinic acid and caffeic acid, the latter of which can be oxidized to caffeic acid quinone (Hapiot et al., 1996). In further support of this idea, caffeic acid itself has been shown to accumulate after *M. incognita* infection in a resistant tomato cultivar but not in three susceptible ones (Afifah et al., 2019). However, since caffeic acid is also involved in lignification (Boerjan et al., 2003), another defense response against nematodes (Sato et al., 2019), this is circumstantial evidence at best. Furthermore, the involvement of chlorogenic acid in nematode resistance is not universal: in the interaction between coffee and *Meloidogyne exigua*, chlorogenic acid accumulated to a similar degree in a susceptible and a resistant cultivar (Machado et al., 2012).

The phenolic plant hormone salicylic acid (SA) could also be included in this section. Although SA shows some nematistatic and nematicidal activity *in vitro* (Wuyts et al., 2006b), it is present in plant roots at concentrations far below those reported to be nematistatic. Instead, SA appears to be involved in nematode resistance *via* its role as a plant hormone. SA signaling is involved in the regulation of various immune responses against nematodes and is involved in genetic (Branch et al., 2004) and induced resistance to root-knot nematodes (Martínez-Medina et al., 2017). Pre-inoculation treatment with SA or chemical analogs thereof enhances plant resistance to subsequent nematode infection, whereas SA-deficient mutants show increased susceptibility (Wubben et al., 2008; Uehara et al., 2010; Martínez-Medina et al., 2017).

#### Stilbenoids and Diarylheptanoids

Stilbenoids and diarylheptanoids are two relatively small classes of plant secondary metabolites derived from the PPP.

The biosynthesis of stilbenoids involves the coupling of a phenylpropanoyl-CoA (e.g., cinnamoyl-CoA or *para*-coumaroyl-CoA) to three malonyl-CoA units through repeated condensation reactions catalyzed by STILBENE SYNTHASE. This gives rise to the basic C6-C2-C6 stilbene skeleton (*trans*-resveratrol if derived from *para*-coumaroyl-CoA; pinosylvin if derived from cinnamoyl-CoA). These basic structures can be further modified to form the various derivatives known as stilbenoids (Jeandet et al., 2010).

The biosynthesis of diarylheptanoids is less well-understood, but is similar to that of stilbenoids in its initial steps. A POLYKETIDE SYNTHASE catalyzes a condensation reaction between a phenylpropanoyl-CoA (e.g., *para*-coumaroyl-CoA) and malonyl-CoA to form a diketide intermediate. After a second condensation reaction between this diketide and another phenylpropanoyl-CoA molecule, a linear diarylheptanoid (C6-C7-C6) is formed. Cyclization of this compound gives rise to the phenylphenalenone backbone. Linear and cyclic diarylheptanoids may undergo further processing, notably through hydroxylation (Brand et al., 2006; Munde et al., 2013).

**Figure 3:**
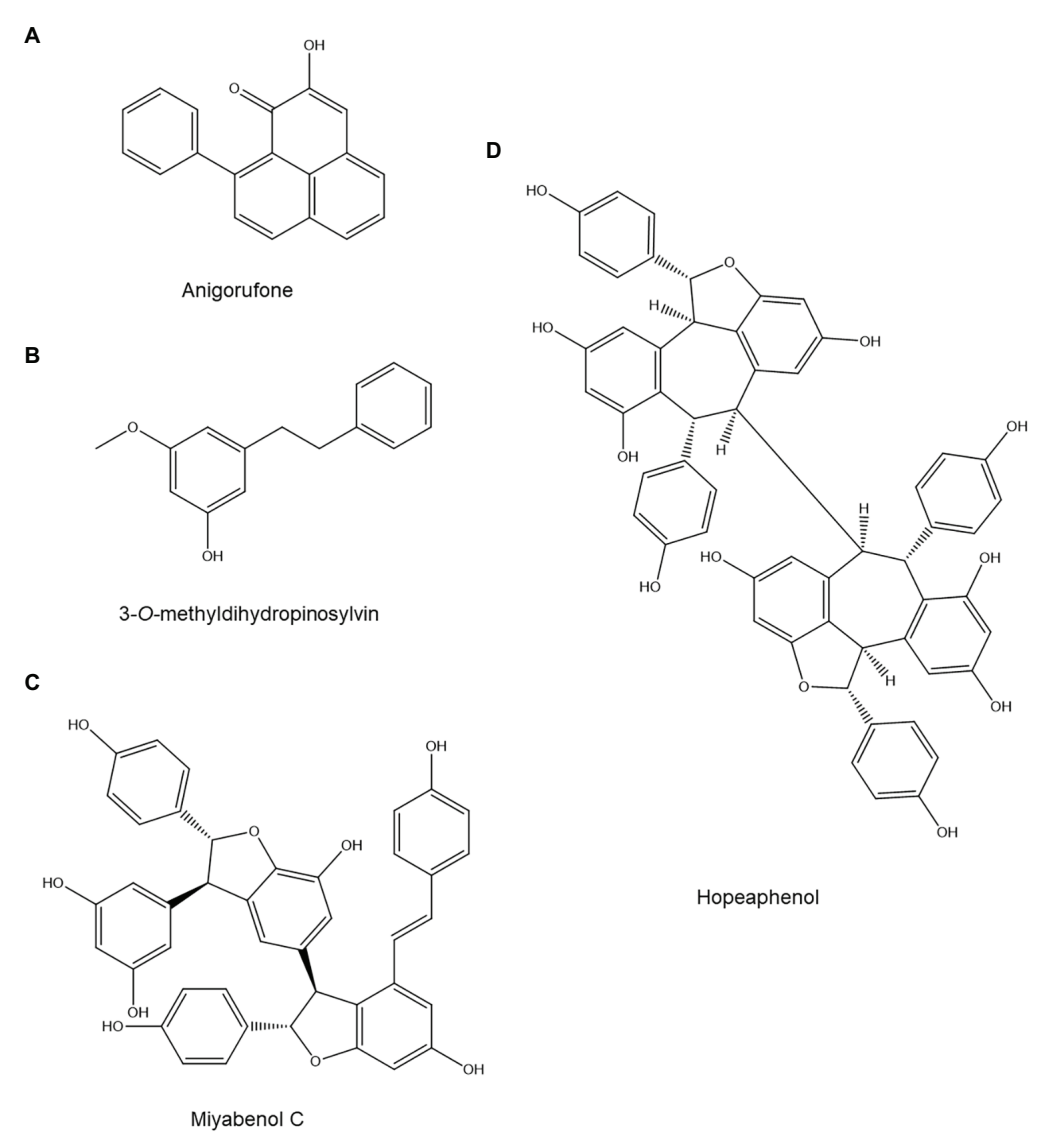
Stilbenoids and diarylheptanoids with possible anti-nematode activity: The phenylphenalenone (cyclic diarylheptanoid) anigorufone **(A)**, the stilbenoid 3-*O*-methyldihydropinosylvin **(B)**, the stilbenoid oligomers miyabenol C **(C)**, and hopeaphenol **(D)**.

Although few studies have examined the role of stilbenoids and diarylheptanoids in plant disease resistance, the available evidence suggests that they are key defense compounds in plants that produce them (Luis, 1998; Echeverri et al., 2012; Akinwumi et al., 2018). Similarly, their role in PPN resistance also appears to be highly significant. Stilbenoids have been implicated in nematode resistance in pine trees and grape vines. The stilbenoid 3-*O*-methyldihydropinosylvin (Figure 3B) accumulated in bark and wood of *Pinus strobus* after infection with the pinewood nematode *Bursaphelenchus xylophilus* and showed significant nematicidal activity *in vitro* (Hanawa et al., 2001). Furthermore, the accumulation of 3-*O*-methyldihydropinosylvin in bark and wood of *P. strobus* coincides temporally with resistance to *B. xylophilus*: the nematode can initially successfully penetrate, but its movement and reproduction stop after approximately 1 week, which is the same time point at which 3-*O*-methyldihydropinosylvin reaches concentrations in bark and wood above those required for nematicidal activity *in vitro*. *In vitro* tests showed that 24 h of exposure to 250 μg/ml of 3-*O*-methyldihydropinosylvin killed 100% of nematodes, while bark and wood of infected *P. strobus* plants accumulated approximately 1,000 and 400 μg/g of this compound. These results, thus, indicate that 3-*O*-methyldihydropinosylvin is a phytoalexin with a major role in *P. strobus* resistance to *B. xylophilus* (Hanawa et al., 2001).

A comparison of two grapevine (*Vitis vinifera*) rootstocks, one of which was susceptible to *M. incognita* and one of which was resistant, also hinted at a possible role for stilbenoids in nematode resistance (Wallis, 2020). The two rootstocks (both with Cabernet Sauvignon as the scion) were inoculated with *M. incognita* and sampled 6 and 12 weeks postinoculation. Throughout the experiment, the resistant and susceptible rootstock showed similar total stilbenoid levels. Furthermore, total stilbenoid content was unaffected by nematode infection in both rootstocks. However, the stilbenoid profile varied significantly between the two rootstocks: the stilbenoid trimer miyabenol C (Figure 3C) and the stilbenoid tetramer hopeaphenol (Figure 3D) were 4–10 times more abundant in the resistant rootstock. These compounds might act as phytoanticipins against PPN, but their anti-nematode activity was unfortunately not assessed *in vitro*.

Phenylphenalenone phytoalexins have been shown to be key players in banana resistance to the burrowing nematode *Radopholus similis*. When banana roots were collected 12 weeks after nematode inoculation, these cyclic diarylheptanoids were found to be significantly more abundant near *R. similis* infection sites in resistant banana varieties than in the susceptible reference cultivar (Hölscher et al., 2014). Out of 13 phenylphenalenones that could be identified in extracts from the resistant banana cultivars, three showed significant nematistatic activity in an *in vitro* assay (Hölscher et al., 2014). Further investigation on the most abundant of those, anigorufone (Figure 3A), showed that its IC_50_ on *R. similis* motility was 59 and 23 μg/ml after 24 and 72 h, respectively (Hölscher et al., 2014). The researchers showed that anigorufone forms complexes with lipids inside the nematode, leading to the formation of large lipid-anigorufone droplets and eventual nematode death (Hölscher et al., 2014). Attempts to quantify anigorufone *in planta* found concentrations of approximately 39 mg/g root tissue in and around *R. similis* infection sites, which shows that banana roots accumulate biologically relevant anigorufone concentrations (Hölscher et al., 2014).

#### Flavonoids

Flavonoids are the largest family of phenolic secondary metabolites, with more than 10,000 identified members (Mathesius, 2018). Flavonoids have long been implicated in plant resistance to pests and diseases other than PPN (Treutter, 2005, 2006) and are also among the most widely studied plant secondary metabolites in relation to PPN resistance. This extensive body of research is reflected in the length of this section, which significantly exceeds those on other metabolite classes.

Flavonoid biosynthesis starts similarly to that of stilbenoids, but the first committed step is catalyzed by CHALCONE SYNTHASE rather than STILBENE SYNTHASE. Both enzymes share high sequence homology, and STILBENE SYNTHASE is believed to have evolved from CHALCONE SYNTHASE (Tropf et al., 1994). CHALCONE SYNTHASE condenses *para*-coumaroyl-CoA with three malonyl-CoA units to form a chalcone skeleton, which then undergoes isomerization to form the corresponding flavonoid. Flavonoids may then be further processed through e.g., hydroxylation, methylation, prenylation, and glycosylation (Naoumkina et al., 2010; Falcone Ferreyra et al., 2012). Depending on the flavonoid backbone, flavonoids are subdivided into bioflavonoids (2-phenylchromen-4-one skeleton), isoflavonoids (3-phenylchromen-4-one skeleton), and neoflavonoids (4-phenylcoumarin skeleton; McNaught and Wilkinson, 1999).

*In vitro* experiments have shown that several common flavonoids show (limited) anti-nematode activity: kaempferol is inhibitory to the hatching of *R. similis* eggs while kaempferol, quercetin, and myricetin are both repellent and somewhat nematistatic (but not nematicidal) to *M. incognita* juveniles (Wuyts et al., 2006b). The effect of flavonoids on nematode behavior, however, is complex: they can either attract or repel *M. incognita* juveniles depending on their molecular structure and concentration (Kirwa et al., 2018). Flavonoids have been most extensively studied in relation to plant-nematode interactions in the Fabaceae family, whose members produce various isoflavonoids and pterocarpans (phytoalexins derived from isoflavonoids *via* coupling of the isoflavonoid B ring and 4-one position) in response to infection.

**Figure 4:**
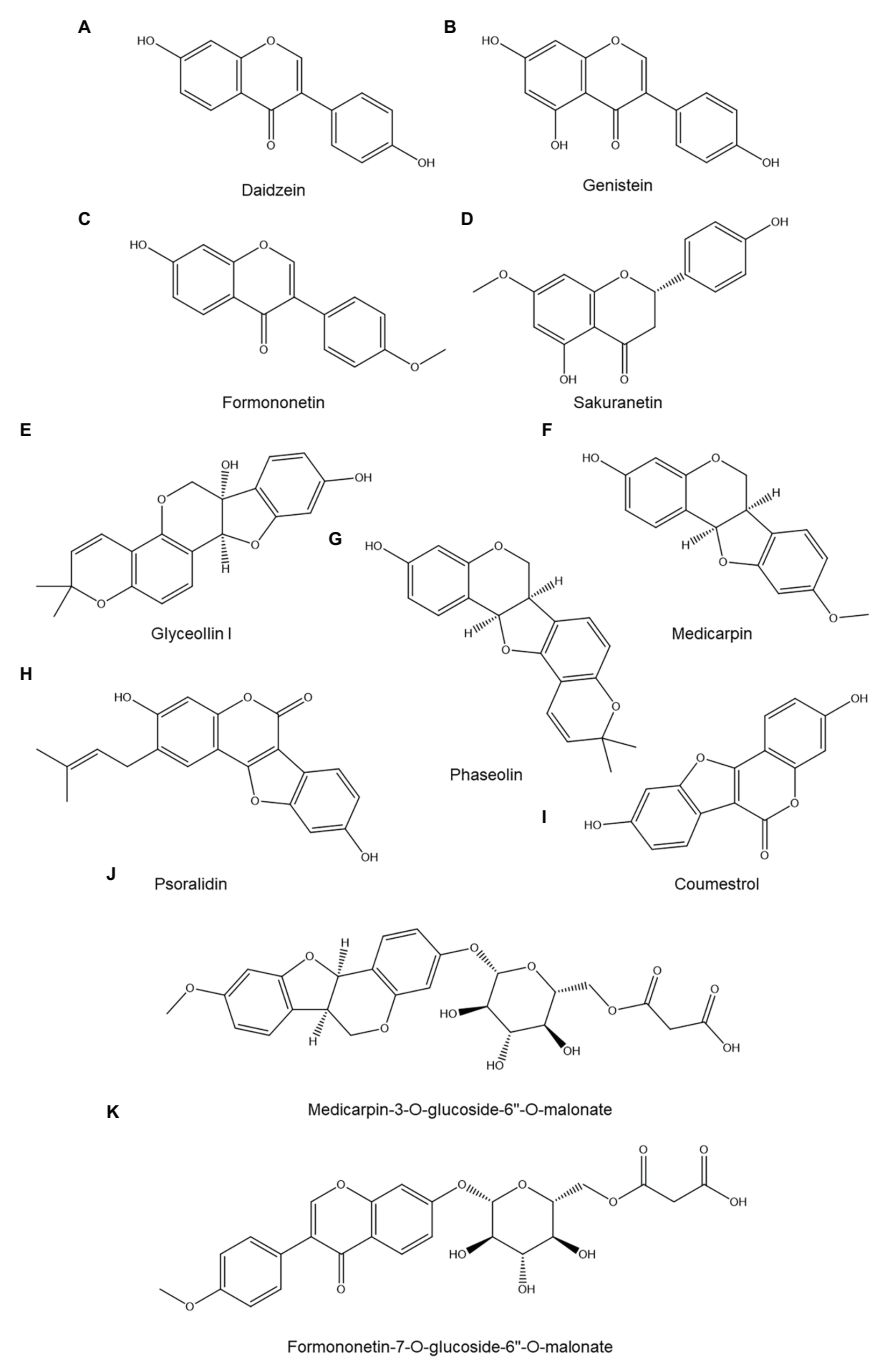
Overview of identified flavonoids discussed in this review: daidzein **(A)**, genistein **(B)**, formononetin **(C)**, sakuranetin **(D)**, glyceollin I **(E)**, medicarpin **(F)**, phaseolin **(G)**, psoralidin **(H)**, coumestrol **(I)**, medicarpin-3-*O*-glucoside-6''-*O*-malonate **(J)**, and formononetin-7-*O*-glucoside-6''-*O*-malonate **(K)**.

A well-studied pterocarpan in relation to nematode resistance is the phytoalexin glyceollin I (Figure 4E), produced by soybeans (*Glycine max*). Glyceollin I accumulated near the head region of soybean cyst nematodes (*H. glycines*) as soon as 8 h post penetration in a resistant soybean cultivar but not in a susceptible one (Huang and Barker, 1991). It was undetectable in roots prior to nematode infection but gradually accumulated afterward. Glyceollin I levels peaked 4–6 days post penetration and declined afterward; in the resistant cultivar root, Glyceollin I levels reached 23 μg/g of fresh root, whereas the susceptible cultivar accumulated three times less and showed no preferential accumulation near the nematode head (Huang and Barker, 1991). The authors argue that the preferential deposition near the head of the nematode is indicative of an elicited response (Huang and Barker, 1991); the molecular pattern(s) in the head region of *H. glycines* that are responsible for this remain unidentified.

Similarly, glyceollin I may also play a role in soybean resistance to *M. incognita* (Kaplan et al., 1980a). While the concentration of glyceollin I in roots of a resistant cultivar reached 80 μg/g of fresh root 7 days postinoculation, a susceptible variety accumulated five times less. Furthermore, there was a clear spatiotemporal correlation between glyceollin I accumulation and the occurrence of a hypersensitive response (HR) in the resistant cultivar: both glyceollin accumulation and HR began around 3 days postinoculation and glyceollin I concentrations were highest in the root stele, the only root tissue where HR was observed. Whether HR and glyceollin I accumulation are independent resistance mechanisms or interact in some way remains unclear. The authors also reported that when the *M. incognita*-resistant cultivar was instead inoculated with *Meloidogyne javanica*, a related nematode species to which it is not resistant, glyceollin I did not accumulate, which further supports the hypothesis that glyceollin I deposition is a specific, induced resistance response.

The mechanism of action of glyceollin I against nematodes has been partially elucidated: *in vitro* motility assays showed that biologically relevant concentrations of glyceollin I are strongly nematistatic to J2 juveniles of *M. incognita* and inhibit their respiration (Kaplan et al., 1980a,b) but had no effect on *M. javanica* juveniles (Kaplan et al., 1980a). The precise target(s) of glyceollin I within the nematode remains unknown.

In contrast to glyceollin, the isoflavonoids daidzein (Figure 4A) and genistein (Figure 4B) – the most abundant flavonoids in soybean roots and their exudates – appear to play no significant role in soybean resistance to *H. glycines*, as both flavonoids accumulated to a similar degree in a susceptible and a resistant cultivar after nematode infection (Kennedy et al., 1999). This observation is supported by the absence of *in vitro* nematicidal activity of these isoflavonoids toward *R. similis* (although a repellent effect was observed; Wuyts et al., 2006b).

Accumulation of the pterocarpan phaseolin (Figure 4G) has been reported in the roots of susceptible common bean (*Phaseolus vulgaris*) seedlings 5 days after penetration by *Pratylenchus penetrans*, reaching an estimated concentration of 59 μg/g of fresh root. However, *in vitro* exposure to a similar phaseolin concentration had no effect on PPN motility or survival, which makes it unlikely that phaseolin accumulation is a major contributor toward defense against *P. penetrans* (Abawi and Vanetten, 1971).

By contrast, the pterocarpan medicarpin (Figure 4F) did show nematistatic effects against *P. penetrans*, with an IC_50_ just below 20 μg/ml (Baldridge et al., 1998). Resistant alfalfa (*Medicago sativa*) accessions showed significantly higher constitutive expression of several genes involved in isoflavonoid biosynthesis compared to susceptible cultivars, but high-performance liquid chromatography (HPLC) analysis revealed that all varieties contained similar total isoflavonoid levels both before nematode inoculation and for at least the 2 subsequent days. Furthermore, there was no correlation between basal medicarpin concentration and nematode resistance among the tested cultivars (Baldridge et al., 1998). Based on these results, medicarpin and other isoflavonoids appear to be at most minor contributors to alfalfa resistance to nematodes.

The roles of medicarpin and the isoflavonoid formononetin (Figure 4C) as well as their malonated glycosides medicarpin-3-*O*-glucoside-6''-*O*-malonate (Figure 4J) and formononetin-7-*O*-glucoside-6''-*O*-malonate (Figure 4K) in resistance to the stem nematode *Ditylenchus dipsaci* has been studied in white clover (*Trifolium repens*; Cook et al., 1995). A resistant and susceptible white clover variety contained similar basal levels of all four metabolites in roots, leaves, and meristems, but after *D. dipsaci* inoculation, the resistant variety began to accumulate more medicarpin and formononetin in the inoculated meristems. This accumulation began relatively late in the infection process: it was not yet visible 3 days postinoculation but was significant 7 and 10 days postinoculation. The glycosylated forms accumulated to similar degrees in susceptible and resistant plants. Neither the resistant nor the susceptible variety showed systemic flavonoid accumulation: increased flavonoid levels were only observed in the meristem. The direct effects of medicarpin and formononetin on *D. dipsaci* were not examined, so to what extent – if any – these flavonoids contribute to resistance remains unclear (Cook et al., 1995).

Work by the same group on alfalfa showed that neither resistant nor susceptible alfalfa accumulates additional isoflavonoids in aerial tissues after *D. dipsaci* infection, but that the resistant cultivar showed a two- to three-fold increase in root isoflavonoid content (Edwards et al., 1995). The reason for this phenomenon is unclear, but the authors note that *D. dipsaci* predisposes alfalfa to root infection by *Fusarium* wilt and bacterial wilt. Based on this, they speculate that accumulation of defense metabolites in roots might be an adaptive response to prevent secondary infections (Edwards et al., 1995).

Coumestans, oxidized pterocarpans, appear to be significant phytoalexins in plant-nematode interactions. When Lima beans (*Phaseolus lunatus*) and common beans were exposed to the nematode *Pratylenchus scriberni*, to which Lima beans are resistant whereas common beans are susceptible, it was found that Lima beans accumulated substantial quantities of two coumestans tentatively identified as coumestrol (Figure 4I) and psoralidin (Figure 4H; Rich et al., 1977). Basal levels of coumestrol were similar between Lima and common beans, whereas basal psoralidin levels were two times higher in Lima beans. Two days after infection, coumestrol levels in common beans remained nearly unchanged, whereas those in Lima bean roots had increased more than three-fold. Similarly, psoralidin concentrations were unresponsive to inoculation in common bean but increased over two-fold in Lima bean. Both coumestrol and psoralidin primarily accumulated in Lima bean in and around sites of HR, where these compounds reached concentrations 7–32 times above their *in vitro* IC_50_ toward *P. scriberni* motility (10–15 μg/ml; Rich et al., 1977).

A handful of studies have examined the role of flavonoids in interactions between cereals and nematodes. When shoots of various rice (*Oryza sativa*) varieties were sampled 5 days after inoculation with the stem nematode *Ditylenchus angustus*, a resistant rice variety was found to contain 13 μg/g of fresh weight of the flavonoid phytoalexin sakuranetin (Figure 4D), whereas this compound was not found in any of the susceptible varieties that were analyzed (Gill et al., 1996). This result is entirely correlative, since the effects of sakuranetin on *D. angustus* were not investigated.

In oat (*Avena sativa*), Soriano et al. (2004) observed a two- to three-fold increase in the shoot and root concentration of three methanol-soluble compounds with UV-absorbance spectra reminiscent of flavonoids upon infection by *Heterodera avenae* or *Pratylenchus neglectus*, as well as upon foliar treatment with the defense hormone methyl jasmonate. A crude methanol extract of methyl jasmonate-induced oat was highly nematicidal toward *H. avenae*. Two of the three inducible flavonoid phytoalexins could be purified, and one of them was strongly nematicidal. The inducible flavonoids were eventually partially identified as three flavone-*C*-glycosides: an apigenin-*C*-hexoside-*O*-pentoside (not nematicidal), an *O*-methyl-apigenin-*C*-deoxyhexoside-*O*-hexoside (nematicidal), and a luteolin-*C*-hexoside-*O*-pentoside (could not be purified).

Induction of these flavonoids by treating plants with methyl jasmonate 3 days prior to inoculation significantly reduced the total nematode population 10 days postinoculation for both *H. avenae* and *P. neglectus* and increased the percentage of nematodes present outside the root rather than inside. A similar effect was seen when susceptible wheat plants were treated with a flavonoid-rich extract from induced oats plants. Taken together, these results indicate that inducible flavonoids from oat are both repellent and nematicidal. However, these flavonoids appear to be only effective *in planta* against *H. avenae* and *P. neglectus* when they are present prior to or shortly after penetration (e.g., *via* methyl jasmonate pre-treatment). In untreated susceptible plants, infection by *H. avenae* or *P. neglectus* eventually caused the concentration of inducible flavonoids to increase to the level seen in methyl jasmonate-induced plants, and yet the nematodes could reproduce normally (Soriano et al., 2004).

By contrast, HPLC-MS analysis of root extracts from several lines of a single-seed descent population derived from a cross between two oats cultivars different to the one used by Soriano et al. (2004) found no correlation between susceptibility to *H. avenae* and the basal concentration of the three flavonoids mentioned previously. Flavonoid accumulation is thus at most one of several resistance mechanisms against *H. avenae* present in oat germplasm (Bahraminejad et al., 2008).

*Arabidopsis thaliana transparent testa (tt)* mutants, which are impaired in the biosynthesis of flavonoids, have been used to study the role of flavonoids in PPN resistance. One study reported that none of the tested *tt* mutants differed from their wild type in susceptibility to *M. incognita* (Wuyts et al., 2006a), while another study found that against *Heterodera schachtii* most *tt* mutants show either unchanged or slightly increased susceptibility compared to their wild types (Jones et al., 2007). These results suggest that flavonoids play at most a minor role in PPN resistance in *A. thaliana*.

Several authors have proposed that sedentary nematodes may exploit flavonoids as part of their pathogenesis process (Chin et al., 2018), based on the observations that PPN alter plant auxin homeostasis during feeding site formation (Grunewald et al., 2009a,b) and that several flavonoids have been described as inhibitors of auxin transport (Ng et al., 2015). However, evidence for this hypothesis is circumstantial at best. If PPN extensively manipulated flavonoids as a pathogenesis strategy, it would be expected that *A. thaliana tt* mutants show increased susceptibility. As mentioned in the previous paragraph, this is not generally the case. In support of the idea that PPN exploit flavonoids, it has been reported that the expression of *CHALCONE SYNTHASE*, a key gene in flavonoid biosynthesis, coincides spatiotemporally with an increased auxin response in developing *M. incognita* feeding sites in white clover (Hutangura et al., 1999) and that a flavonoid-deficient *Medicago truncatula* transgenic line hosts smaller *M. incognita* galls than its wild type (Wasson et al., 2009). Both results are, however, entirely correlative and do not prove that PPN manipulate auxin *via* flavonoids.

The results in this section collectively indicate that the role of flavonoids in PPN resistance depends on the specific flavonoids and nematodes involved, and possibly also on the timing of accumulation in the infection process. That the flavonoid glyceollin I plays a role in soybean resistance toward *H. glycines* and *M. incognita* appears convincingly established, but evidence in other pathosystems remains mixed.

#### Tannins

Tannins are a heterogeneous group of polyphenolic compounds. They are usually divided in two subgroups, the hydrolysable and the condensed tannins. Hydrolysable tannins possess a polyol core to which galloyl groups are esterified, while condensed tannins are oligomers of two or more flavan-3-ols. Both types show an enormous diversity in degree of polymerization, monomer composition and in decoration with other phenolic compounds (Barbehenn and Peter Constabel, 2011; Salminen and Karonen, 2011). They are involved in plant resistance to insect herbivory (Barbehenn and Peter Constabel, 2011; Salminen and Karonen, 2011) and a handful of studies have also found a correlation between tannin accumulation and nematode resistance. However, no causal evidence has been presented, perhaps because obtaining pure and representative tannin standards remains difficult (Barbehenn and Peter Constabel, 2011). Tannins were historically believed to hinder herbivores by inducing protein precipitation and, thus, depriving them of nutrition (Barbehenn and Peter Constabel, 2011; Salminen and Karonen, 2011). However, more recent evidence has shown that this effect may be negligible *in vivo* and that tannins instead derive their activity from cytotoxic and antinutritive products formed when tannins are oxidized by plant POLYPHENOL OXIDASES or by the alkaline gut environment present in many insect herbivores (Barbehenn and Peter Constabel, 2011; Salminen and Karonen, 2011).

In banana, a cultivar resistant to *R. similis* contained a higher basal condensed tannin concentration than susceptible cultivars when analyzed 12 weeks after inoculation (Collingborn et al., 2000). Although condensed tannin levels increased significantly upon nematode infection in all cultivars, their concentration in the susceptible cultivars remained far below that of the resistant variety. The same trend was observed for flavan-3,4-diols, the main precursors of condensed tannins in banana (Collingborn et al., 2000). The resistant banana cultivar also incorporated propelargonidins alongside the usual procyanidin in its condensed tannins (Collingborn et al., 2000); whether the resistance of the banana cultivar can be attributed to its higher tannin concentration and/or its different tannin composition remains unclear, as the direct effects of banana tannins on *R. similis* were not evaluated.

A putative role for tannins in resistance to the pinewood nematode *B. xylophilus* has also been proposed. When *B. xylophilus* was cultured on the phloem sap of eight pine species, its growth rate was negatively correlated to the concentration of condensed tannins in the sap of each species (Pimentel et al., 2017). However, negative correlations were also observed between nematode growth rate and total flavonoid concentration as well as total phenolic compound concentration. This makes it difficult to assess the relative contribution of condensed tannins, flavonoids, and other phenolic metabolites to the inhibitory effect on *B. xylophilus* (Pimentel et al., 2017).

### Terpenoids

Terpenoids, an umbrella term for terpenes and their derivatives, are likely the most diverse class of plant secondary metabolites, with more than 60,000 compounds already identified (Pazouki and Niinemetst, 2016). Terpenes are formed by condensation of two or more activated isoprene units (C5 building blocks), either isopentenyl pyrophosphate or its isomer dimethylallyl pyrophosphate (Cheng et al., 2007). Depending on the number of C5 building blocks involved, this condensation can lead to the formation of a C10 (monoterpene), C15 (sesquiterpene), or C20 (diterpene) terpene. Sesqui- and diterpene units can in turn undergo head-to-head condensation to form C30 (triterpenes, e.g., sterols) or C40 units (tetraterpenes, e.g., carotenoids; Pichersky and Raguso, 2018). All terpenes can be further substituted, e.g., through hydroxylation or acetylation, to form *terpenoids*. Since enzymes acting on terpenoids are both numerous and often highly promiscuous, terpenoids are an exceptionally diverse class of secondary metabolites (Pichersky and Raguso, 2018). Many terpenoids are active against pests and pathogens, and an evolutionary arms race with these attackers may have been a major driver behind the increasing terpenoid diversity seen throughout plant evolution (Pichersky and Raguso, 2018).

**Figure 5:**
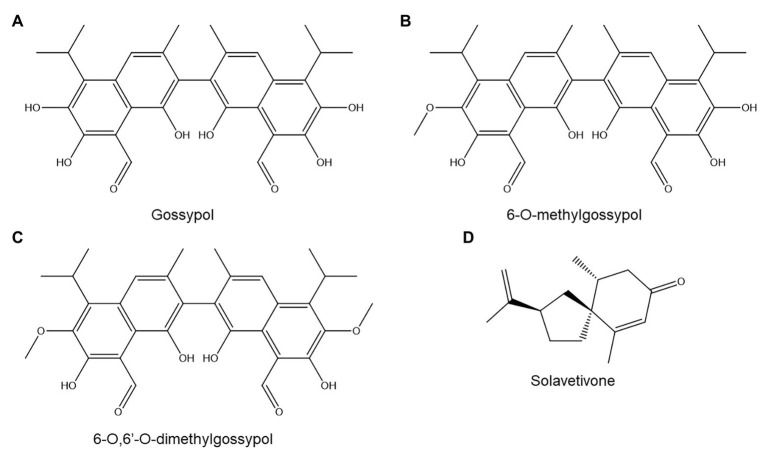
Terpenoids discussed in this review: gossypol **(A)**, 6-*O*-methylgossypol **(B)**, 6-*O*,6'-*O*-dimethylgossypol **(C)**, and solavetivone **(D)**.

The most widely studied group of terpenoids in plant-nematode interactions are the terpenoid aldehydes (TAs) of cotton (*Gossypium* sp.), which include gossypol (Figure 5A) and its derivatives. Gossypol is a polyphenolic compound but is included under terpenoids owing to its biosynthesis: it is formed by oxidative coupling of two repeatedly oxidized sesquiterpene units (Heinstein et al., 1979).

The role of TAs in cotton resistance to *M. incognita* has been extensively studied but remains unclear. One article reported that a resistant upland cotton (*Gossypium hirsitum*) cultivar produced higher basal and induced gossypol levels than a susceptible one, and also exuded gossypol and other TAs to its rhizosphere (Hedin et al., 1984). Histological analysis of TAs in *G. hirsitum* demonstrated that TAs accumulated in root tissues traversed by migrating *M. incognita* juveniles and around their developing feeding sites (Veech, 1979). Although this accumulation occurred both in a susceptible and in two resistant cultivars, the resistant varieties showed significantly earlier accumulation (4 days postinoculation rather than 12–14 days). Furthermore, resistant cultivars showed TA accumulation throughout the entire root, whereas in the susceptible variety, TAs accumulated only in the endodermis and cortex (Veech, 1979).

However, a later study examined three different resistant *G. hirsitum* cultivars and found that two of them had lower basal and induced TA concentrations than two susceptible reference cultivars (Khoshkhoo et al., 1994a). Three possible explanations for this observation were hinted at by the authors: (a) TA accumulation might be one of several resistance mechanisms present in cotton, (b) TAs might play no major role in resistance to *M. incognita*, or (c) the ratio of different TAs rather than total TA concentrations might determine resistance.

**Figure 6:**
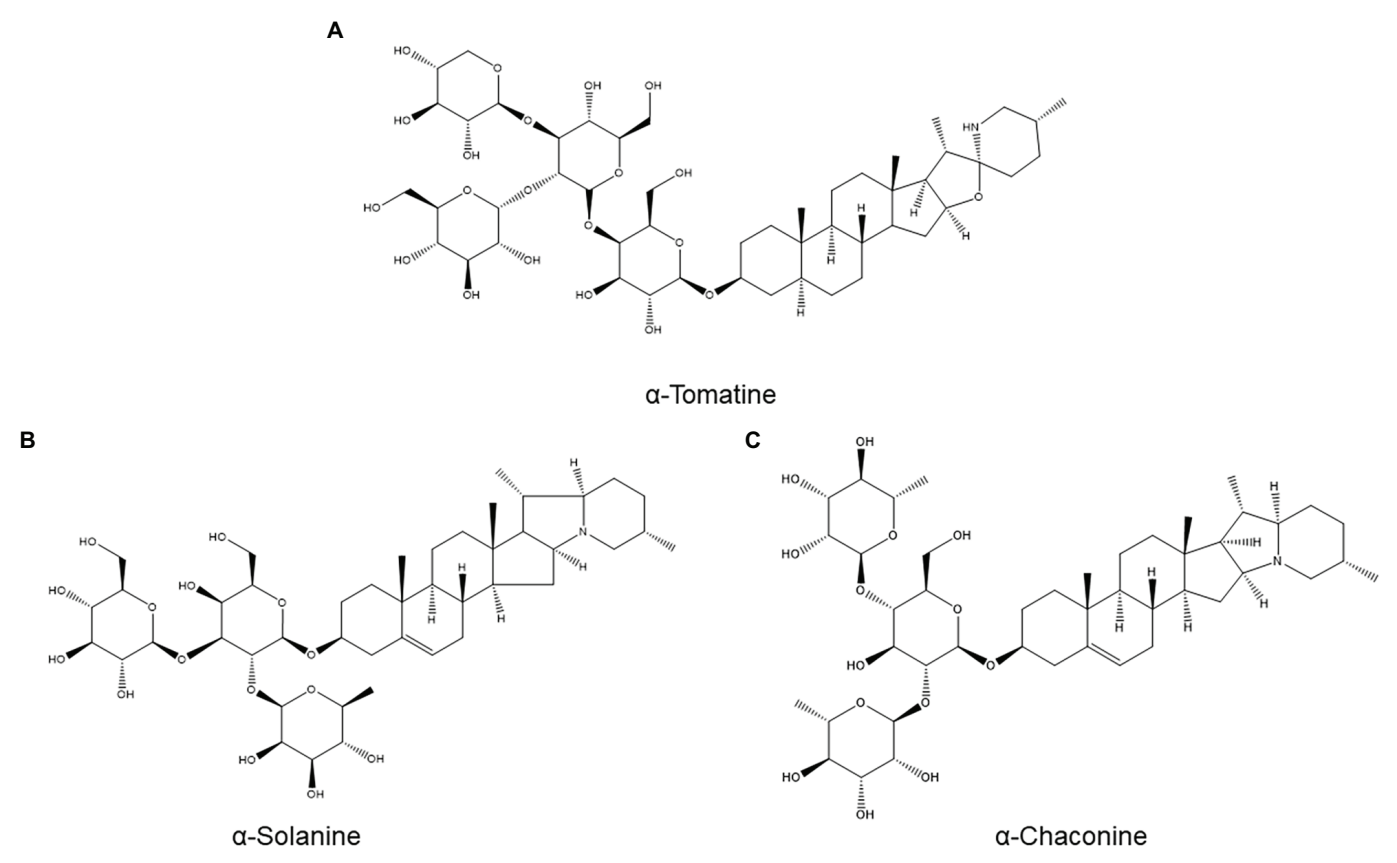
Structures of three saponins investigated for a possible role in nematode resistance: the tomato saponin *α*-tomatine **(A)** and the potato saponins α-solanine **(B)** and α-chaconine **(C)**.

In tentative support of the third hypothesis, another study on five *G. hirsitum* cultivars found no correlation between *M. incognita* resistance and total basal or induced TA concentrations but did find a correlation between resistance and the abundance of one TA sub-class: methylated TAs (Veech, 1978). The author found that the concentration of methylated TAs decreased in two susceptible cultivars 7 days after nematode inoculation, while it rose in three resistant ones (Veech, 1978). The precise structures of the methylated TAs were not provided, but other studies have shown that methylated TAs in cotton root include 6-*O*-methylgossypol (Figure 5B) and 6-*O*,6'-*O*-dimethylgossypol (Figure 5C; Frankfater et al., 2009).

A crude TA mixture, obtained *via* extraction of *G. hirsitum* roots followed by partial purification, showed strong nematistatic activity toward *M. incognita* juveniles with an IC_50_ of 10–50 μg/ml; concentrations upward of 125 μg/ml were also nematicidal (Veech, 1979). Interestingly, an extract from *Gossypium arboreum*, which is believed to produce only unmethylated TAs, showed significantly lower anti-nematode activity than the *G. hirsitum* extract. In turn, a pure gossypol acetate standard was even less nematicidal than the *G. arboreum* extract (Veech, 1979).

Cotton transgenic lines expressing *A. thaliana NPR1*, a gene involved in SA-mediated immunity, showed enhanced resistance to the reniform nematode *Rotylenchulus reniformis* as well as to various fungal pathogens (Parkhi et al., 2010). The *NPR1* lines showed identical basal root TA levels compared to the control but showed enhanced TA accumulation upon infection with the fungal pathogen *Verticillium dahliae*. The authors did not investigate whether this also occurs upon *R. reniformis* infection, and it was also found that *NPR1* expression triggers other defense responses besides TA accumulation (e.g., higher chitinase and glucanase activity). As such, it is hard to attribute a specific role for TAs in resistance to *R. reniformis*.

In pepper (*Capsicum annuum*), the relative concentrations of various terpenes in root exudates in several varieties were correlated with their susceptibility to *M. incognita* (Kihika et al., 2017). Olfactometer tests confirmed that several terpenes exuded by *C. annuum* had repellent or attractive effects on *M. incognita* J2s, which indicates that exuded terpenes may play a role in PPN susceptibility by enhancing or inhibiting host-finding (Kihika et al., 2017).

Solanaceae produce various sesquiterpene phytoalexins, whose role in PPN resistance remains unclear. One study found that potato varieties in which the sesquiterpene solavetivone (Figure 5D) forms a greater than average fraction of total sesquiterpene levels show higher levels of resistance to *Globodera rostochiensis* (Desjardins et al., 1995). However, cultivars with high solavetivone production all shared a common *Solanum tuberosum ssp. andigena* ancestor line (Desjardins et al., 1995), so the resistance of these lines may be caused by another trait inherited from this ancestor line rather than by solavetivone accumulation.

Several plant species produce terpenoid phytoalexins that have not yet been evaluated for a role in nematode resistance. For example, among cereals, maize (*Zea mays*) produces both diterpenoid (kauralexins and dolabralexins) and sesquiterpenoid (zealexins) phytoalexins (Block et al., 2019), while rice produces three different classes of diterpenoid phytoalexins: momilactones, phytocassanes, and oryzalexins (Yamane, 2013). All these terpenoids are involved in defense against fungal, bacterial, and/or insect pests and pathogens (Dillon et al., 1997; Lu et al., 2018; Pichersky and Raguso, 2018; Block et al., 2019), and they are inducible by treatment with resistance inducers that reduce susceptibility to nematodes (Yamane, 2013; Verbeek et al., 2019); it may thus prove fruitful to investigate their role in PPN resistance.

Apart from having direct anti-nematode effects, terpenoids are also involved indirectly in plant-nematode interactions by acting as plant hormones. Abscisic acid is derived from a tetraterpenoid (Nambara and Marion-Poll, 2005), the brassinosteroids from a triterpenoid (Choe, 2006), and the various gibberellins are diterpenoids (Hedden and Thomas, 2012). All of these hormones are known to play varying (often antagonistic) roles in plant resistance to PPN (Nahar et al., 2012, 2013; Kyndt et al., 2017; Bauters et al., 2018; Song et al., 2018; Yimer et al., 2018).

### Saponins

Saponins are plant secondary metabolites defined as glycosides of a C30 terpenoid (Moses et al., 2014). Owing to their terpenoid aglycone, they could have been included in the previous section, but due to their diversity and unique properties (e.g., surfactant activity (Osbourn, 1996)), we opted to place them in a separate subcategory. A major example of saponins with roles in plant defense are the toxic glycoalkaloids produced by various Solanaceae, including *α*-tomatine (Figure 6A) from tomato and α-solanine (Figure 6B) and α-chaconine (Figure 6C) from potato (Osbourn, 1996).

α-Tomatine, which plays a role in tomato resistance to fungal pathogens and insect pests (Elliger et al., 1988), has been evaluated for a role in resistance to *M. incognita* (Elliger et al., 1988). Twelve tomato cultivars with varying resistance levels were assayed for basal root α-tomatine concentrations, and no correlation between α-tomatine production and resistance could be discerned. Furthermore, root α-tomatine concentrations were unchanged by *M. incognita* infection at all examined time points (between 3 and 14 days postinoculation) in both a resistant and a susceptible cultivar. Although it cannot be ruled out that α-tomatine might accumulate selectively near infection sites, which might be missed by bulk root analysis, these data indicate that α-tomatine is unlikely to be a major ANP (Elliger et al., 1988).

One study speculatively linked the resistance of the wild potato species *Solanum canasense* to *Globodera pallida* to its high glycoalkaloid content but provided no evidence for this hypothesis (Castelli et al., 2006). Moreover, in cultivated potato, no correlation was found between tuber glycoalkaloid content (α-solanine and α-chaconine) and resistance to *G. pallida* or *G. rostochiensis* in a breeding population of potato lines with glycoalkaloid contents ranging from 50 to 1,680 mg/kg (Grassert and Lellbach, 1987).

The absence of a role for glycoalkaloids in resistance to potato cyst nematodes is supported by another study, which compared *G. pallida* resistance and root glycoalkaloid content in four potato lines (two cultivated potato varieties and two progenies from a cross between cultivated potato and the *G. pallida*-resistant wild potato *Solanum vernei*; Forrest and Coxon, 1980). There was no correlation between resistance and either basal glycoalkaloid content or glycoalkaloid content 1 month after nematode inoculation. In fact, the most resistant accession had the lowest glycoalkaloid content (Forrest and Coxon, 1980).

In line with these results, alfalfa saponins also do not appear to play a role in nematode resistance: no correlation was found between resistance to *Meloidogyne hapla* and *D. dipsaci* and basal saponin concentration in six alfalfa cultivars (Pedersen et al., 1976).

In oats, however, saponins have been tentatively linked to *H. avenae* resistance. HPLC-MS analysis of fractionated extracts from root tips of single-seed descent lines with varying levels of resistance to *H. avenae* found three peaks whose abundance was highly correlated to *H. avenae* resistance (Bahraminejad et al., 2008). Two of these subfractions contained compounds partially characterized as avenacins, a class of saponins common in oats. The third fraction contained an unstable metabolite that could not be characterized further (Bahraminejad et al., 2008). Although this result is correlative, it suggests that these avenacins might be anti-nematode phytoanticipins.

Overall, there is currently little evidence to suggest that saponins play a major role in plant resistance to nematodes. However, based on the limited range of host plants in which saponins were studied and on the known nematicidal effects of several saponin-rich plant extracts (Chitwood, 2002; Ntalli and Caboni, 2012), it seems premature to draw firm conclusions on the importance of saponins in PPN resistance.

### Alkaloids

Alkaloids are an extremely heterogenous group of plant secondary metabolites whose only commonality is that they contain at least one nitrogen atom, often in a heterocyclic ring. Most, but not all, alkaloids are ultimately derived from amino acids. Some authors have proposed the idea that true alkaloids are defined by a common biosynthesis mechanism involving the formation of a Schiff base followed by a Mannich condensation, rather than by a common precursor or structure (Waterman, 1998).

While the taxonomy of alkaloids is complicated and ambiguous, their importance in the plant kingdom is clear. True alkaloids have been found in over one-fifth of all plant species, and over 12,000 unique alkaloids have been identified (Ziegler and Facchini, 2008; Schläger and Dräger, 2016). Many are highly toxic and play important roles in plant defense against pests and pathogens (Mithöfer and Boland, 2012).

**Figure 7:**
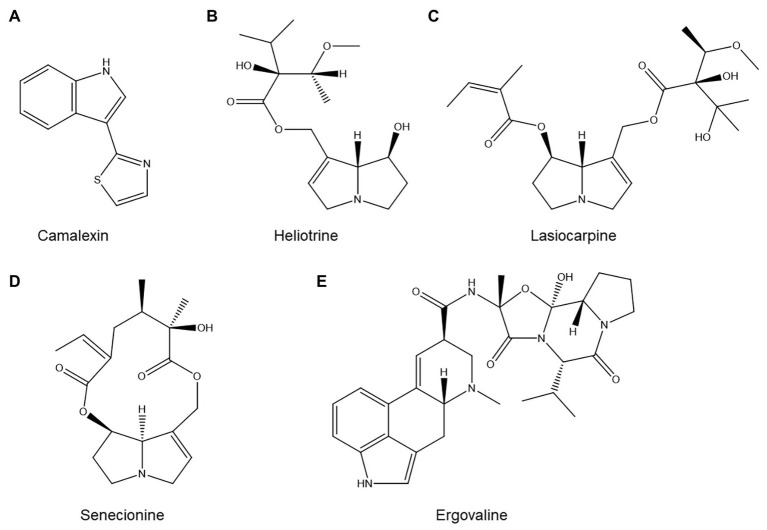
Structures of several alkaloids with possible roles in nematode resistance: **(A)** the *Arabidopsis thaliana* alkaloid camalexin; **(B–D)** three common pyrrolizidine alkaloids with nematicidal activity; and **(E)** ergovaline, one of the ergot alkaloids produced by endophytic fungi of *Pratylenchus*-suppressive grasses.

Camalexin (Figure 7A) is the primary phytoalexin of the model plant *A. thaliana*. This small tryptophan-derived indole alkaloid has been extensively studied and is implicated in *A. thaliana* resistance to a broad spectrum of pests and diseases (Zook and Hammerschmidt, 1998). The *cyp79b2/b3* double mutant, which is severely impaired in camalexin production, showed a statistically significant increase in *H. schachtii* reproduction compared to its wild type (Shah et al., 2015). Similarly, the *pad3* mutant, which is also impaired in camalexin biosynthesis, was significantly more susceptible to *M. incognita* (Teixeira et al., 2016).

The role of nicotine, the principal alkaloid of tobacco (*Nicotiana tabacum*), in resistance to *M. incognita* has been investigated (Davis and Rich, 1987). A resistant tobacco cultivar accumulated significantly more nicotine 4 days after inoculation, whereas the concentration of nicotine in the susceptible cultivar remained unchanged. Whether this plays a causal role in *M. incognita* resistance is unclear: even the susceptible cultivar reportedly contained basal nicotine concentrations that were nematicidal *in vitro* (Davis and Rich, 1987).

Pyrrolizidine alkaloids, a widespread alkaloid family, also play a role in PPN resistance. The available literature on this topic has already been thoroughly reviewed by Thoden and Boppré (2010), so we will not attempt to duplicate this excellent work. Briefly, the authors observe that plants which accumulate substantial amounts of pyrrolizidine alkaloids in their roots are generally very poor hosts for PPN. Moreover, both pure pyrrolizidine alkaloid standards and extracts from pyrrolizidine alkaloid-rich plants tend to be nematicidal *in vitro*, and soil amendments composed of pyrrolizidine alkaloid-rich plants suppress PPN. For illustrative purposes, three common pyrrolizidine alkaloids with known nematicidal activity (Thoden et al., 2009) are shown in Figures 7B–D.

Interestingly, not all alkaloids found in plants are produced by the plant themselves. Notably, grasses often contain ergot alkaloids produced by endophytic fungi (e.g., ergovaline, shown in Figure 7E). Ergot alkaloids may play a role in resistance to *Pratylenchus* sp., based on the observations that grasses colonized by endophytic fungi such as *Epichloë* spp. suppress *Pratylenchus* in field conditions and that ergot alkaloids are nematicidal *in vitro* (Bush et al., 1997; Bacetty et al., 2009). However, one study showed that in at least one grass-endophyte system, knocking out alkaloid biosynthesis in the fungus did not eliminate its ability to induce resistance to *P. scriberni* in perennial ryegrass (*Lolium perenne*; Panaccione et al., 2006). Alkaloid production is thus at most one of several mechanisms by which fungal endophytes enhance the nematode resistance of their hosts.

### Benzoxazinoids

Benzoxazinoids are secondary metabolites with a key role in defense against various insect pests (Niemeyer, 2009; de Bruijn et al., 2018) that are most commonly – but not exclusively – found in grasses, including in cereals such as wheat (*Triticum aestivum*), rye (*Secale cereale*), and maize (Niemeyer, 2009; de Bruijn et al., 2018). All benzoxazinoids are derived from indole, which undergoes a series of steps involving hydroxylation, ring expansion, methoxylation, and glycosylation to yield the various benzoxazinoids (Gierl and Frey, 2001).

**Figure 8:**
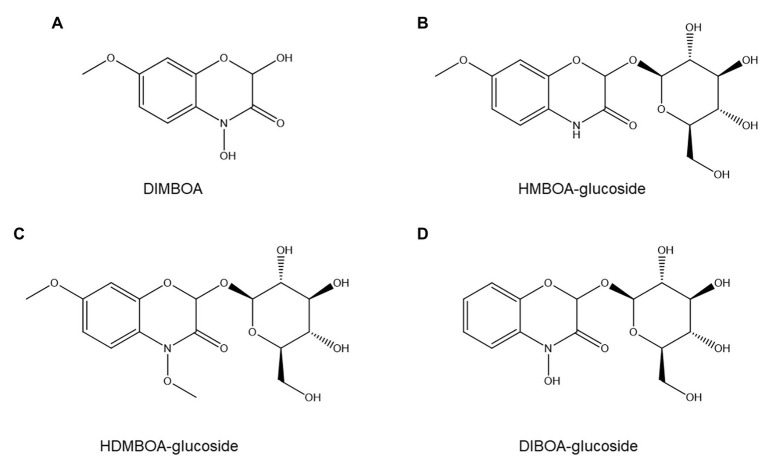
Structures of four common benzoxazinoids evaluated for possible roles in nematode resistance: DIMBOA **(A)**, HMBOA-glucoside **(B)**, HDMBOA-glucoside **(C)**, and DIBOA-glucoside **(D)**.

The importance of benzoxazinoids in PPN resistance has not been extensively studied but appears to be modest at most. No correlation was found between benzoxazinoid content and resistance to the stubby-root nematode *Paratrichodorus minor* in 12 maize hybrids (Timper et al., 2007), nor between levels of the principal maize benzoxazinoid DIMBOA (Figure 8A) and resistance to *P. penetrans* (Friebe, 2001). Furthermore, DIMBOA at the concentration present in maize root exudates was an attractant for *P. penetrans* (Friebe, 2001). Although several rye benzoxazinoids show nematistatic activity *in vitro* (Zasada et al., 2005), their concentration in roots and root exudates is too low to have ANP activity (Meyer et al., 2009).

In contrast to these negative results, another study reported that inoculating wheat with an arbuscular mycorrhizal fungus that repressed benzoxazinoid production significantly increased susceptibility to *P. neglectus* (Frew et al., 2018). Furthermore, a wheat cultivar with higher susceptibility to *P. neglectus* showed lower basal and induced benzoxazinoid concentrations (Frew et al., 2018). Individual benzoxazinoids showed contrasting accumulation patterns between treatments: HMBOA-glucoside (Figure 8B) and HDMBOA-glucoside (Figure 8C) appeared positively correlated to resistance to *P. neglectus*, whereas this was not observed for DIBOA-glucoside (Figure 8D; Frew et al., 2018).

### Glucosinolates

Plants in the order Brassicales produce varying levels of glucosinolates, metabolites derived from glucose and an amino acid which are characterized by the presence of both sulfur and nitrogen atoms. Enzymatic hydrolysis of glucosinolates by MYROSINASE releases unstable, biocidal isothiocyanates with a major role in resistance to insects and plant pathogens (Rask et al., 2000; van Dam et al., 2009).

*In vitro* data suggest that glucosinolates have strong nematicidal activity and biofumigation, the use of Brassica seed meal or green manures as soil amendments, can be an effective alternative to chemical fumigation (van Dam et al., 2009). However, the evidence available on their *in vivo* role in resistance to nematodes remains limited.

**Figure 9:**
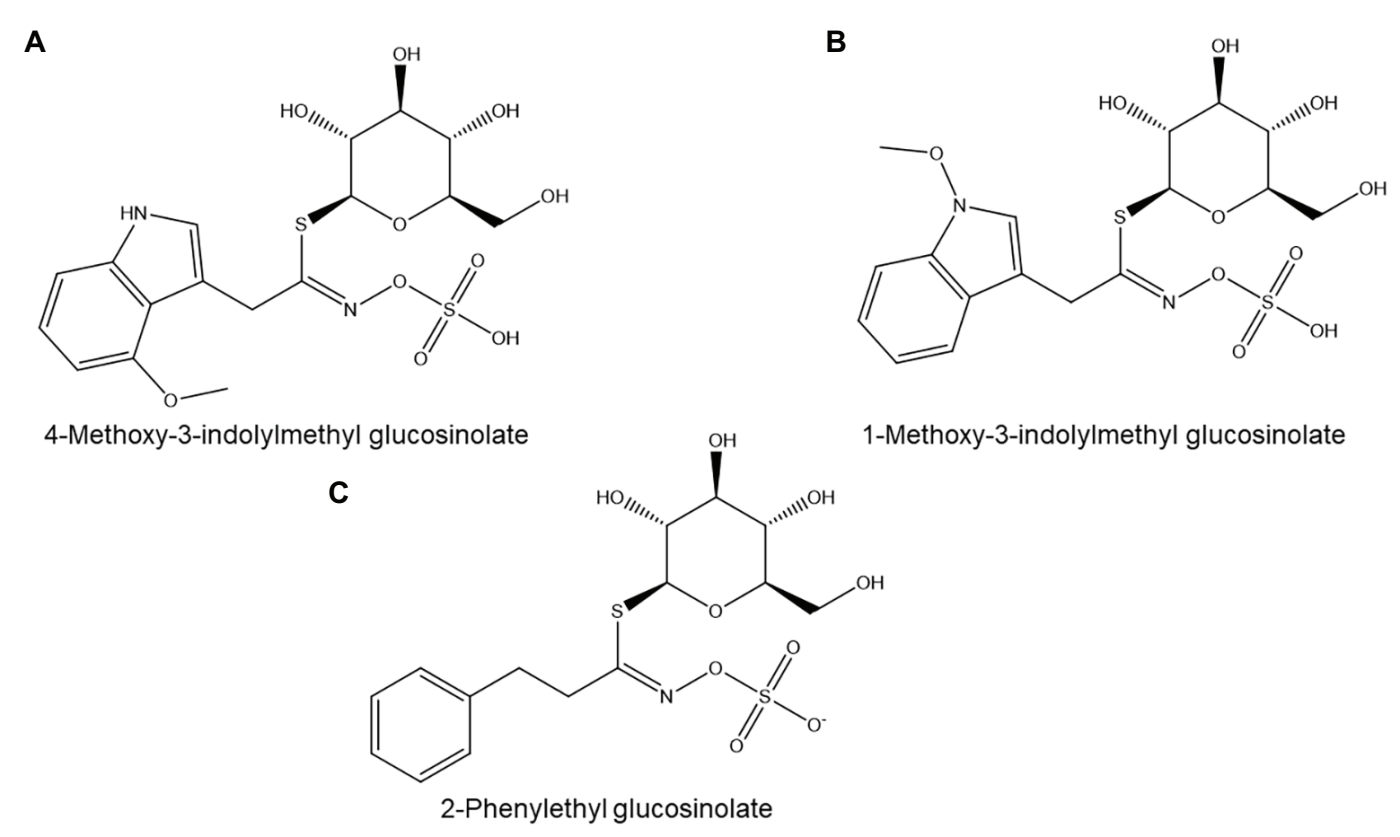
Glucosinolates with possible nematicidal activity: **(A,B)** Two major indolic glucosinolates from *A. thaliana*. **(C)** 2-Phenylethyl glucosinolate, a major glucosinolate from *Brassica napus*.

In a survey of *Brassica napus* accessions, susceptibility to *P. neglectus* proved uncorrelated to total glucosinolate content (Potter et al., 1999). However, a clear relationship was found between susceptibility to *P. neglectus* and the concentration of one specific glucosinolate, 2-phenylethyl glucosinolate (Figure 9C). All cultivars producing more than a critical threshold estimated to be between 8 and 12 μmol/g fresh root tissue showed low susceptibility to *P. neglectus*. However, other resistance mechanisms must also exist in *B. napus*, as several accessions with low 2-phenylethyl glucosinolate content also showed low susceptibility (Potter et al., 1999). The efficacy of various *Brassica* species as biofumigants against *P. neglectus* was also correlated with their 2-phenylethyl glucosinolate content but not with their total glucosinolate concentration (Potter et al., 1998).

By contrast, a comparison of the susceptibility of 11 Brassicaceae species toward *M. javanica* found no correlation between resistance and either total glucosinolate content or glucosinolate composition (McLeod et al., 2001).

In *Arabidopsis thaliana*, the *myb34/51* double mutant, which is impaired in the biosynthesis of indolic glucosinolates such as 4-methoxy-3-indolylmethyl glucosinolate (Figure 9A) and 1-methoxy-3-indolylmethyl glucosinolate (Figure 9B), showed significantly higher susceptibility to *M. incognita* than its wild type (Teixeira et al., 2016). *MYB34* expression is also significantly downregulated in giant cells of *M. incognita*-infected *A. thaliana*, which provides further support for a role for glucosinolates in PPN resistance (Portillo et al., 2013).

### Organosulfur Compounds

**Figure 10:**
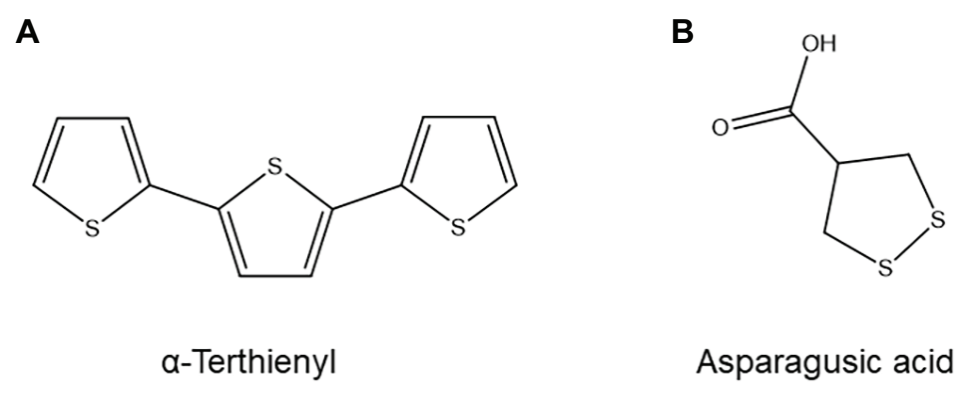
Structure of two nematicidal organosulfur compounds: **(A)** α-Terthienyl, one of several nematicidal polythienyl compounds produced by marigolds. **(B)** Asparagusic acid, a nematicidal asparagus metabolite.

The resistance of several marigolds (*Tagetes* sp.) toward PPN and the suppressive effect of *Tagetes* cultivation on nematode populations have been attributed to strongly nematicidal polythienyl compounds present in *Tagetes* roots and their exudates, such as *α*-terthienyl (Figure 10A; Uhlenbroek and Bijloo, 1958; Chitwood, 2002). A meta-analysis of 175 Asteraceae species found that their suppressive effect on *P. penetrans* populations was highly correlated with their polythienyl content: out of 16 Asteraceae species known to produce α-terthienyl, 15 were suppressive to *P. penetrans* (Gommers and Voorin’tholt, 1976). An unidentified acetylenic dithio compound with a bright red coloration was also correlated with anti-PPN activity: 11 out of 12 evaluated Asteraceae species known to exude this compound were suppressive to *P. penetrans* (Gommers and Voorin’tholt, 1976).

The biosynthesis of α-terthienyl and related compounds remains to be fully elucidated, although several mechanisms have been suggested (Arroo et al., 1995). The nematicidal mechanism of action of α-terthienyl, by contrast, has been studied in more detail than that of most other ANPs. It has been demonstrated that α-terthienyl generates reactive oxygen species upon activation by light and/or peroxidase enzymes (Chitwood, 2002; Hamaguchi et al., 2019). In *Caenorhabditis elegans*, RNAi-lines with suppressed or induced accumulation of SUPEROXIDE DISMUTASE and GLUTATHIONE PEROXIDASE respectively showed increased and reduced susceptibility to α-terthienyl. Furthermore, α-terthienyl readily penetrated the nematode hypodermis (Hamaguchi et al., 2019). These results indicate that α-terthienyl owes its nematicidal effect to its ability to induce oxidative stress inside the nematode (Hamaguchi et al., 2019).

Asparagus (*Asparagus officinalis*), another plant with nematode-suppressive properties, was shown to produce a highly nematicidal compound in its roots that could be identified as the organosulfur compound asparagusic acid (Figure 10B; Takasugi et al., 1975). Asparagusic acid at a concentration of 50 ppm has strong nematicidal properties against several PPN species and inhibits *Heterodera* egg hatching (Takasugi et al., 1975). Since asparagus roots were found to contain at least 35 ppm of asparagusic acid, it is likely that this compound is a phytoanticipin with a major role in the anti-nematode activity of asparagus plants (Takasugi et al., 1975). Asparagusic acid biosynthesis is believed to be unique to asparagus and is poorly understood, but likely involves isobutyric acid and methacrylic acid as precursors and the amino acid cysteine as the donor of at least one of the sulfur atoms (Mitchell and Waring, 2014).

## Identifying ANPs: Methods, Challenges, and Recent Advances

Identification of ANPs remains a challenging task, despite technological advances. Historically, most ANPs were identified by preparing crude extracts from nematode-suppressive or resistant plants, either with or without elicitation by nematode inoculation, and then laboriously (sub)-fractionating these extracts until pure compounds, or more frequently mixes of a few related compounds, were obtained. By assaying these (sub-)fractions for anti-nematode efficacy, an active (sub)-fraction could be identified. This fraction was then subjected to a range of analytical methods such as elemental analysis, ultraviolet/visible light (UV/VIS) and infrared spectroscopy, color reagents, and, in more recent studies, mass spectrometry (MS) or nuclear magnetic resonance (NMR). These techniques often allowed the researcher(s) to propose candidate compounds, which were then synthesized chemically and used as analytical standards. This approach is extremely laborious and requires large quantities of input material, often upward of 20 kg (Uhlenbroek and Bijloo, 1958; Takasugi et al., 1975).

Not all researchers who identified ANPs had to start from scratch. In some cases, plant metabolites that had previously been identified as being involved in plant resistance to other plant pests or pathogens were deliberately investigated for a role in PPN resistance. Because these potential ANPs were known in advance, extraction, and quantification could proceed in a targeted, more rapid manner. This “shortcut” facilitated e.g., the identification of glyceollin I as a key player in soybean PPN resistance (Kaplan et al., 1980b).

Further evidence for a causal role occasionally came from histopathological methods, which can show whether metabolites of interest preferentially accumulate at or near PPN infection sites. This approach was used to demonstrate that glyceollin I accumulated near the head of *H. glycines* in resistant soybean (Huang and Barker, 1991) and that terpenoid aldehydes accumulate more rapidly and widely in RKN-affected root areas in resistant cotton cultivars (Veech, 1979).

A more modern, hitherto relatively uncommon approach, for evaluating the role of metabolites in nematode resistance involves infection experiments in mutants impaired in the biosynthesis of these metabolites. If the mutant in question is thoroughly characterized and free of interfering pleiotropic effects (e.g., because the biosynthesis of related products of the same pathway is also eliminated), this method can yield strong evidence for a causal role in resistance. Unfortunately, generating mutants requires either thorough knowledge of the biosynthetic pathways involved in the production of a metabolite (in which case targeted mutagenesis can be used), or an extremely laborious process of random mutagenesis followed by metabolic or phenotypic screening. Furthermore, transformation protocols remain unavailable for many non-model plant species. By consequence, extensive libraries of mutants exist only in a limited number of model plants and pathways. Despite these drawbacks, mutant analysis has been successfully employed to study e.g., the role of *A. thaliana* secondary metabolites in nematode resistance (Shah et al., 2015; Teixeira et al., 2016).

Another method for putative ANP identification involves assembling a panel of different cultivars or closely related species with varying susceptibility to a certain PPN and then trying to correlate this variation in resistance to basal or induced levels of a specific metabolite (Giebel, 1970; Hung and Rohde, 1973; Veech, 1978; Hedin et al., 1984; Grassert and Lellbach, 1987; Elliger et al., 1988; Gill et al., 1996; Baldridge et al., 1998; Potter et al., 1999). This approach has three major downsides: it depends on the proper selection of a sufficiently diverse and representative panel to avoid false positives, it cannot prove causality and it requires a possible ANP to be known in advance. However, the latter downside can be avoided by the emergence of a novel analytical approach: untargeted metabolomics.

Untargeted metabolomics is a collective term for methods that seek to provide an unbiased, comprehensive picture of the metabolite composition of a biological sample. Through untargeted profiling of plant varieties or species with varying degrees of resistance to a PPN, metabolites with possible roles in nematode resistance may be identified and studied further. As well as requiring little prior knowledge, modern metabolomics methods also require only small quantities of input material (often just 100 mg of fresh plant material). However, much larger quantities of material may still be required later on to allow the extraction and purification of sufficient quantities of putative ANP for bioassays.

Untargeted metabolomic analysis of plant-nematode interactions remains uncommon but has been more widely used to study plant interactions with bacteria, fungi, and insects (Heuberger et al., 2014; Feussner and Polle, 2015; Maag et al., 2015; Tenenboim and Brotman, 2016; van Dam and Bouwmeester, 2016). In relation to the study of ANPs, the objective of untargeted metabolomics is to identify metabolites which discriminate resistant and susceptible plants, either basally or after nematode infection. Such metabolites can then be evaluated for their possible role as ANPs using the various techniques described previously, such as testing their *in vitro* anti-nematode activity or examining the effect of knock-out mutants in their biosynthesis on nematode resistance.

The handful of metabolomics studies performed on plant-nematode interactions are discussed in the remainder of this review.

Gas chromatography-mass spectrometry (GC-MS) based profiling has been used to study the interaction between the sting nematode *Belonolaimus longicaudatus* and three bermudagrass (*Cynodon transvaalensis*) lines with varying levels of susceptibility (Willett et al., 2020). All lines showed extensive metabolomic reprogramming due to nematode parasitism when they were analyzed 3 months after inoculation, but there were substantial differences between lines and between individuals within each line. Nematode-mediated suppression of amino acid levels in the host plants was found to be highly correlated with higher susceptibility, whereas accumulation of L-pipecolic acid, D-glucuronic acid, glycolate, and phenylalanine correlated with lower susceptibility (Willett et al., 2020). The *in vivo* effect of these metabolites was not investigated, so their ANP status remains putative. However, L-pipecolic acid is a known inducer of systemic immunity in plants (Shan and He, 2018; Wang et al., 2018), while phenylalanine is the principal precursor of the phenylpropanoid pathway, which has an important role in plant immunity *via* the biosynthesis of phenolic phytoalexins and lignin (Vogt, 2010).

Another study combined untargeted GC-MS based metabolomics and transcriptomics on soybean roots inoculated either with *H. glycines*, a plant-growth promoting bacterium (PGPB) known to induce partial resistance to *H. glycines* or both (Kang et al., 2018). The authors identified four metabolites which were suppressed in plants 5 days after inoculation with *H. glycines* alone but not in plants co-inoculated with the PGPB and *H. glycines*. These metabolites were the phenolic compound 4-vinylphenol, the alkaloid piperine, the amino acid L-methionine, and the fatty acid palmitic acid. All four showed *in vitro* nematicidal effects at concentrations upward of 500 μg/ml, but no indication was given of the concentration of these metabolites *in planta* (Kang et al., 2018). As such, it is impossible to judge their importance to *H. glycines* resistance.

GC-MS based metabolic profiling of 5, 10, and 15 days-old syncytia in a compatible interaction between *H. schachtii* and *A. thaliana* showed that *H. schachtii* parasitism induces extensive reprogramming of primary metabolism, notably of amino acid and oligosaccharide metabolism (Hofmann et al., 2010). Since the study examined only a single, susceptible cultivar, no candidate ANPs could be identified; however, the oligosaccharides and amino acids identified as being affected by nematode parasitism might be targets for further research.

Combined transcriptome and metabolome (using HPLC-MS/MS) profiling of mature *M. incognita* galls (21 days postinoculation) in poplar roots showed that, compared to uninfected root tissue of the same age, *M. incognita* galls show severe disruption of genes and metabolites involved in cell wall biosynthesis and phenolic metabolism. Interestingly, although galls generally appeared to accumulate greater amounts of phenolic compounds than uninfected roots, chlorogenic acid was among the most strongly repressed metabolites in galls (Baldacci-Cresp et al., 2020).

Untargeted HPLC-MS analysis has been used to identify possible biomarkers for oat resistance to *H. avenae* (Bahraminejad et al., 2008). From a population of 170 single-seed descent lines originating from a cross between two oat cultivars, 15 highly resistant, and 15 highly susceptible lines were selected and grown in a misting chamber (without nematode inoculation). Extracts from the root tips of these lines were subjected to HPLC-MS analysis; for each observed peak, the correlation coefficient between its abundance in each line and that line’s resistance to *H. avenae* was calculated. This led to the identification of three peaks whose abundance in uninoculated seedlings was highly correlated to resistance. Attempts to elucidate the structures of the compounds corresponding to these peaks led to the identification of two saponins with sterol cores corresponding to avenacin A-1 and avenacin B-1 but with different (unidentified) glycosides. The third peak contained a compound too unstable for purification. The authors note that HPLC-MS screening is significantly faster and less laborious than resistance trials, and that at least in this breeding population, the three peaks might, after further validation, serve as biomarkers for resistance (Bahraminejad et al., 2008).

HPLC-UV and NMR-based profiling of root extracts from cotton varieties that were either resistant or susceptible to *M. incognita* found only minor differences in the total abundance of flavonoids, gossypol, and gossypol derivatives between the two cultivars when they were sampled at 8, 24, and 35 days after inoculation. However, the resistant cultivar showed significantly higher basal contents of several minor flavonoids and gossypol derivatives that could not be conclusively identified (Alves et al., 2016).

Although MS is best known as a technique for identifying and quantifying metabolites in extracts, MS can also be used as an imaging technique to localize metabolites in plant tissues (a process known as MS imaging, or MSI). An elegant application of this method to study plant-nematode interactions can be found in Hölscher et al. (2014). The authors took NMR spectra of four subfractions derived from a crude ethanol extract made from *R. similis* lesions in a resistant and susceptible banana variety. Based on these spectra, it was found that the resistant cultivar accumulated significantly larger amounts of several identifiable phenylphenalenone compounds. Sections of root areas showing *R. similis* lesions from a susceptible and resistant banana variety were then subjected to UV-laser desorption/ionization MSI, which revealed that metabolites with *m*/*z*-values corresponding to several of the identified phenylphenalenones accumulated in and near lesions – especially in the resistant cultivar. Several phenylphenalenones were isolated from banana root extracts and were found to be strongly nematicidal. Finally, MSI and Raman microspectroscopy revealed that phenylphenalenone anigorufone exerted nematicidal activity by inducing the formation of large lipid-anigorufone complexes inside the nematode’s body. This study demonstrates how metabolomics technologies such as NMR and MS can be used to facilitate all stages of ANP discovery: NMR allowed identification and quantification of possible ANPs, while MS imaging could prove that the localization of the ANP correlated with nematode infection sites and provided information on the ANP’s mechanism of action.

NMR has also been used as a standalone metabolomics technique in several studies on plant-nematode interactions, as will be shown in the following paragraphs.

A combination of NMR and UV/VIS-spectrophotometric assays was used to metabolically profile the roots of a *Meloidogyne exigua*-susceptible and resistant coffee cultivar in the presence or absence of nematode infection at 1, 2, and 4 days after inoculation (Machado et al., 2012). The analysis identified the accumulation of phenolic compounds, sucrose, and fumaric acid as being possibly involved in *M. exigua* resistance. Among phenolic compounds, the abundance of chlorogenic acid was unchanged; however, one of its constituent parts, quinic acid, was significantly more abundant in the resistant cultivar. Amino acids levels, total carbohydrate concentration, and total alkaloid concentration appeared uninvolved in resistance (Machado et al., 2012).

Untargeted NMR-based metabolomics has also been applied to the tomato-*M. incognita* interaction. Root tissue from four cultivars (two highly susceptible and two highly resistant) was collected 38 days after inoculation and analyzed; the researchers found several metabolites that accumulated significantly more after infection in the resistant accessions but not in the susceptible ones. Two of these compounds were conclusively identified as caffeic acid and glucose respectively (Afifah et al., 2019). However, no causal evidence for a role for caffeic acid or glucose in resistance was presented.

NMR metabolomics has also been used to study nematode interactions in seedlings of the soursop tree (*Annona muricata*), which is highly resistant to nematode infection (Machado et al., 2019). Extracts of root systems of soursop seedlings with or without *M. javanica* inoculation were harvested at various time points between 1 and 30 days after inoculation, analyzed through NMR and then used for bio-assay guided fractionation. The experiments revealed that soursop root extracts were nematistatic, and that after fractionation, this activity was concentrated in the chloroform fraction. Further NMR analysis of this fraction showed that it contained several acetogenins, a class of secondary metabolites common among the Annonaceae that have known insecticidal activity. No major metabolome shift was seen in nematode-inoculated plants compared to uninoculated plants at time points later than 2 days postinoculation, which supports the notion that the soursop seedlings were highly resistant and that nematodes could not successfully invade (Machado et al., 2019).

Although untargeted metabolomics is a powerful tool for the identification of novel putative ANPs, progress is hindered by the difficulty of high-throughput metabolite identification. A browse through the PlantCyc metabolite database (Schläpfer et al., 2017) shows that even for relatively well-annotated plant species such as tomato, rice, and *A. thaliana*, between 2,500 and 3,000 metabolites are present and the PlantCyc database as a whole contains fewer than 5,000 unique, characterized metabolites. These values can be compared to the estimated 200,000 distinct secondary metabolites believed to be present in higher plants (Viant et al., 2017).

Another challenge in metabolomics studies is the difficulty of obtaining truly representative, unbiased metabolome profiles. Depending on the choice of sample preparation method and/or analytical technique, different parts of the metabolome will be captured in more detail than others (Ernst et al., 2014; Sumner et al., 2015). Although fully discussing the relative merits of different metabolomics methods is beyond the scope of this review, it is worth mentioning that each of the analytical methods described in this review has biases.

NMR provides the most comprehensive structural information about metabolites (including stereochemistry), is highly reproducible, and is not biased toward metabolites of certain sizes or polarities (Moco et al., 2007; Kim et al., 2010, 2011; Schripsema, 2010). However, it is less sensitive than MS and the absence of a separation step prior to analysis often leads to spectra with strong signal overlap in which few metabolites can be identified (Moco et al., 2007; Kim et al., 2010, 2011; Schripsema, 2010). The sensitivity of NMR has improved over the years, but the problem of spectral overlap remains (Moco et al., 2007; Kim et al., 2010, 2011; Schripsema, 2010). Three main solutions exist for this problem: using 2D-NMR methods (Moco et al., 2007; Kim et al., 2010, 2011; Schripsema, 2010), coupling NMR to LC (Moco et al., 2007; Sumner et al., 2015), or fractionating the extract prior to NMR analysis (Moco et al., 2007; Kim et al., 2010). LC-NMR has not yet been used in studies of PPN, whereas fractionation and 2D NMR have been used with some success by e.g., Machado et al. (2019).

GC-MS and HPLC-MS are widely used metabolomics techniques. Both methods share high sensitivity and excellent separation but differ in other important respects. GC-MS uses high-energy ionization, which leads to detailed, reproducible MS spectra that allow straightforward matching to biological databases (Ernst et al., 2014). However, GC-MS can only detect metabolites which are sufficiently volatile, either natively or after chemical derivatization (Moco et al., 2007; Ernst et al., 2014). This limitation restricts its usefulness to smaller metabolites, whereas ANPs are often relatively large. HPLC-MS, by contrast, is not restricted to volatile compounds but has drawbacks of its own: a single LC column cannot be used to separate metabolites of widely different polarity, and metabolite identification in HPLC-MS may be complicated owing to the relatively poor reproducibility of mass spectra and matrix effects such as the formation of adducts (Ernst et al., 2014). The former disadvantage, however, is usually manageable as most classes of plant secondary metabolites believed to be involved in plant-nematode interactions are semipolar and thus amenable to analysis on a common reversed-phase C18 column (Moco et al., 2007).

Given the differing strengths and weaknesses of NMR, GC-MS and LC-MS, choosing an appropriate technique in advance is important. A common strategy to aid in method selection is to use transcriptome analysis to identify pathways possibly involved in nematode resistance prior to metabolome analysis. Based on the expected size and polarity of the metabolites of pathways identified through transcriptomic analysis, the most appropriate metabolome technique can be chosen. A good example of this approach is found in Baldacci-Cresp et al. (2020): after RNA-seq had shown that the expression of genes involved in phenolic metabolism was severely affected by *M. incognita* parasitism, the authors chose HPLC-MS/MS as the technique best suited to analyzing these metabolites (Baldacci-Cresp et al., 2020).

Despite the challenges involved in choosing an appropriate sample preparation method and analytical technique, metabolomics approaches show clear potential in elucidating the role of secondary metabolites in nematode resistance. As exemplified by the studies mentioned in this review, untargeted metabolomics can identify novel candidate-ANPs in plants, even in non-model species such as *A. muricata* or *C. transvaalensis*.

## Possible Applications of ANPs in PPN Control

Although the identification of novel ANPs has scientific value in itself, ANP discovery also has practical applications. Perhaps the most obvious is to facilitate the discovery of novel botanical nematicides. The nematicidal properties of isothiocyanates have been exploited for decades, either through chemical methyl isothiocyanate precursors such as metam sodium or dazomet (Chitwood, 2003) or *via* biofumigation (van Dam et al., 2009). Various phytochemical-based nematicides, obtained either through extraction or chemical synthesis, are commercially available (Chitwood, 2003; Ladurner et al., 2014; Medico et al., 2018). Novel ANPs, identified through untargeted metabolomics, may be good lead compounds for the development of novel phytochemical-based nematicides.

Identifying ANPs could also provide targets for crop improvement through genetic engineering, either by increasing the biosynthesis of ANPs already present in a species or by enabling the biosynthesis of novel ANPs through transgenic constructs. Although attractive in theory, no such genetically modified crops have been reported to the best of our knowledge.

Finally, ANPs could act as biomarkers in nematode resistance breeding, where high-throughput targeted metabolomics could be used for screening breeding lines instead of laborious nematode resistance assays. Several articles cited in this review have raised the idea of using metabolic markers in resistance breeding.

Terpenoid aldehyde levels in roots, leaves, and seeds have all been evaluated as biomarkers for cotton resistance to *M. incognita* but with limited success: as discussed previously, the correlation between root TA levels and *M. incognita* is imperfect (Khoshkhoo et al., 1994a) and seed and leaf TA content appear to have no predictive value at all (Khoshkhoo et al., 1994b). High root 2-phenylethyl glucosinolate content has been suggested as a possible biomarker for *Pratylenchus* resistance in canola, although here too some highly resistant genotypes were found to contain low root 2-phenylethyl glucosinolate concentrations (Potter et al., 1999). Tannins have been proposed as biomarkers for *R. similis* resistance in banana (Collingborn et al., 2000). Finally, concentrations of flavonoids (Soriano et al., 2004) and saponins (Bahraminejad et al., 2008) in oat roots have been suggested as biomarkers for *H. avenae* resistance, but once again this approach is complicated by the existence of resistance mechanisms independent of flavonoids against *H. avenae* (Bahraminejad et al., 2008). Since multiple resistance mechanisms appear to exist in most breeding pools, metabolic markers must be thoroughly evaluated for each breeding population to avoid an excessively high false negative rate. However, this may be worthwhile in certain cases due to the laborious and time-consuming nature of conventional nematode resistance screening.

Metabolic markers could also be used to screen for induced resistance. Resistance inducers are exogenously applied chemicals or microbes which stimulate the plant immune response against pests or pathogens (Heil, 2001; Eyles et al., 2010; Pieterse et al., 2014; Mauch-Mani et al., 2017), including PPN (Oka et al., 1999; Oka and Cohen, 2001; Molinari and Baser, 2010; Vos et al., 2013; Fujimoto et al., 2015; Ji et al., 2015; Huang et al., 2016; Zhan et al., 2018; Singh et al., 2019). If metabolic markers can be reliably correlated to induced resistance in a given plant-PPN system, they can be used to screen for novel resistance inducers and to study the longevity of induced resistance in a more high-throughput manner than possible through conventional inoculation experiments.

The emergence of untargeted metabolomics paired with high-dimensional statistical methods may enable selection based on *metabolic profiles* rather than based on specific metabolic markers. In this approach, multiple features, each representing a (possibly unidentified) metabolite associated with PPN resistance, are assessed simultaneously to predict PPN resistance. This approach has been successfully demonstrated in breeding for traits such as resistance to fungal pathogens (Hamzehzarghani et al., 2005; Fernie and Schauer, 2009; Saito and Matsuda, 2010; Tomita et al., 2017) but has not yet been applied to plant-nematode interactions. However, the oat-*H. avenae* cases mentioned in this review hint at the possible utility of this approach: since resistance mechanisms involving flavonoids (Soriano et al., 2004) and saponins (Bahraminejad et al., 2008) have been identified, metabolic profiling for both classes of metabolites – and possibly others – will be required for reliable screening.

## Conclusion and Future Perspectives

Since the middle of the twentieth century, a substantial number of studies have proven that plants make extensive use of small molecules to defend against PPN. Although the enormous diversity of metabolites, plants, and nematode species studied to date makes generalization difficult, several conclusions can be drawn from the available literature.

First and foremost, the number of ANPs for which a causal role in resistance to nematodes has been conclusively determined remains highly limited. This may, at least, partially be explained by the difficulty of proving causality in ANP studies. In our opinion, conclusively proving a causal role for an ANP in PPN resistance requires four lines of evidence. First, it must be demonstrated that the abundance of a compound or class of compounds is *correlated* with resistance – e.g., by demonstrating that its abundance is higher in resistant varieties. Second, purified or chemically synthesized candidate-ANPs should show anti-nematode activity *in vitro*. Third, the suspected ANP should accumulate *in planta* to a biologically relevant concentration in or near a site of interaction with the nematode. Finally, reducing or abolishing the production of the ANP in a resistant plant (e.g., through gene silencing or chemical inhibition) should diminish resistance. No single study cited in this review presents all four lines of evidence, although several provide the first three. The rarity of the fourth step may be explained by the fact that many studies cited in this review pre-date the -omics era.

This brings us neatly to a second observation: many of the studies cited in this review are relatively old. Indeed, the average age of original research articles on ANPs cited in this review is nearly 25 years. The apparently declining attention given to ANPs in recent decades is also reflected in reviews on plant immunity to nematodes: whereas reviews on plant resistance to nematodes from the 1980s (Giebel, 1982; Veech, 1982) had ANPs as their primary focus, more recent reviews address them briefly (Sato et al., 2019) or do not mention them at all (Holbein et al., 2016). We believe that revisiting ANPs using the novel research methods that have emerged over the last two decades, such as targeted mutagenesis, transcriptomics, and metabolomics, may be a fruitful way to advance plant nematology.

A third conclusion is that several studies cited in this review report that the total concentration of a certain class of secondary metabolites is uncorrelated to PPN resistance, whereas the concentration of one or more specific, often low-abundance, metabolites within that class does show a strong correlation with resistance. Examples of this phenomenon in this review are found among terpenoid aldehydes in cotton (Veech, 1978, 1979; Hedin et al., 1984; Khoshkhoo et al., 1994a; Alves et al., 2016), flavonoids in soybean (Kaplan et al., 1980a,b; Huang and Barker, 1991; Kennedy et al., 1999; Carpentieri-Pipolo et al., 2005; Kang et al., 2018), stilbenoids in grapevine (Wallis, 2020), benzoxazinoids in wheat (Frew et al., 2018), and glucosinolates in canola (Potter et al., 1998, 1999). This observation stresses the importance of using analytical approaches that identify individual metabolites (e.g., HPLC- or GC-MS) instead of relying on less discriminatory techniques such as colorimetric assays.

Finally, a methodological shift in the study of ANPs can be discerned over time. When the study of ANPs began in the 1950s, researchers tended to work in a targeted manner: ANPs were sought in a low-throughput manner in extracts from plants that were known to have nematode-suppressive properties, or by studying metabolites whose role in resistance to other pests or diseases had already been established. As the performance and accessibility of GC/LC-MS and NMR increased, it became possible to work in a less targeted manner. Although untargeted studies of plant-nematode interactions remain uncommon, they have already enabled the identification of a handful of putative novel ANPs.

Taken together, these last two observations point to the importance of metabolomics approaches – which can be untargeted and independent of prior knowledge, and which examine individual metabolites – in identifying novel ANPs. The recent publication of a handful of metabolomics studies on plant-nematode interactions suggests that researchers are indeed beginning to harness the power of metabolomics. Despite their limited number, these studies have already hinted at novel ANPs in several plant species. This early success may lead to a revival of interest in ANP research, a trend that will doubtlessly be enhanced by the rapid growth of biological databases and the development of more user-friendly data analysis tools (e.g., MetaboAnalyst; Chong et al., 2019). Together with other -omics era research techniques, metabolomics may facilitate the arrival of a “golden age” in ANP research in the coming decade.

## Author Contributions

WD wrote this review with substantial input from SM, TK, and BV. All authors read and approved the final manuscript.

### Conflict of Interest

The authors declare that the research was conducted in the absence of conflicts of interest. Although WD’s PhD grant is co-funded by a private sector partner, Eastman, this review does not touch on subjects of commercial relevance to Eastman, and Eastman and its employees were not involved in this review.
